# Unwinding process of DNA/RNA quadruplexes by proteins under label-free nanopore monitoring

**DOI:** 10.1093/nar/gkaf547

**Published:** 2025-06-26

**Authors:** Meili Ren, Ting Weng, Liyuan Liang, Xun Chen, Daixin Liu, Shaoxi Fang, Rong Tian, Wanyi Xie, Liang Wang, Deqiang Wang, Chunyu Zeng

**Affiliations:** Chongqing Institute of Green and Intelligent Technology & Chongqing School, University of Chinese Academy of Sciences, Chongqing 400714, PR China; Department of Cardiology, The First Affiliated Hospital of Kunming Medical University, Kunming 650032, Yunnan Province, PR China; Chongqing Institute of Green and Intelligent Technology & Chongqing School, University of Chinese Academy of Sciences, Chongqing 400714, PR China; Chongqing Institute of Green and Intelligent Technology & Chongqing School, University of Chinese Academy of Sciences, Chongqing 400714, PR China; Chongqing Institute of Green and Intelligent Technology & Chongqing School, University of Chinese Academy of Sciences, Chongqing 400714, PR China; Chongqing Institute of Green and Intelligent Technology & Chongqing School, University of Chinese Academy of Sciences, Chongqing 400714, PR China; Chongqing Institute of Green and Intelligent Technology & Chongqing School, University of Chinese Academy of Sciences, Chongqing 400714, PR China; Chongqing Institute of Green and Intelligent Technology & Chongqing School, University of Chinese Academy of Sciences, Chongqing 400714, PR China; Chongqing Institute of Green and Intelligent Technology & Chongqing School, University of Chinese Academy of Sciences, Chongqing 400714, PR China; Chongqing Institute of Green and Intelligent Technology & Chongqing School, University of Chinese Academy of Sciences, Chongqing 400714, PR China; Chongqing Institute of Green and Intelligent Technology & Chongqing School, University of Chinese Academy of Sciences, Chongqing 400714, PR China; Chongqing Institute of Green and Intelligent Technology & Chongqing School, University of Chinese Academy of Sciences, Chongqing 400714, PR China; Department of Cardiology, Daping Hospital, Third Military Medical University, Chongqing 400000, PR China; Key Laboratory of Geriatric Cardiovascular and Cerebrovascular Disease Research, Ministry of Education of China, Chongqing Key Laboratory for Hypertension Research, Cardiovascular Clinical Research Center, Chongqing Institute of Cardiology, Chongqing 400000, PR China; Department of Cardiology, The First Affiliated Hospital of Kunming Medical University, Kunming 650032, Yunnan Province, PR China

## Abstract

Noncanonical quadruplexes (G4s) in the nucleic acids represent specific secondary structures that correlate and participate in important biological processes, including telomeric propagation and tumor cell proliferation, and are close to the life span. The interaction of G4 with specific proteins and monitoring of the unfolding process are important to understand the development and evolution of some diseases and for further regulation of telomere and to disclose the mechanism of typical cancers. Different from the most reported single-molecule tweezers’ manipulation, this work provides a nanopore-based electric approach for label-free monitoring of the unfolding process of both DNA- and RNA-G4 under various conditions. Twenty nanomolar of hTel sequence could be mostly unfolded via incubation with 10 nM of both TEP1 that is associated with telomerase and helicase RTEL1 under weak acidic conditions for 1 h, and this process could be recorded in a single-molecule nanopore device with a pore diameter of 3.7 nm in 0.5 M CsCl buffered solution at 150 mV. TEP1 is proved to specifically interplay with hybrid G4 and nearly does not unfold parallel G4. The efficient and selective unfolding process of RNA-G4 originated from SARS-CoV by helicase nsp13 is also demonstrated at pH 5 in 2 M LiCl with a 3.6-nm pore; with 50% molar ratio of helicase nsp13, RNA-1574-G4 could be selectively and significantly unfolded in 1 h. This work presents the selective unfolding of both DNA- and RNA-G4 by specific proteins, which is a new approach to modulate and monitor the secondary structure of nucleic acids, and shed light on the understanding of the mechanism of the interaction between nucleic acids and proteins and the related kinetics.

## Graphical Abstract

**Graphical Abstract:**
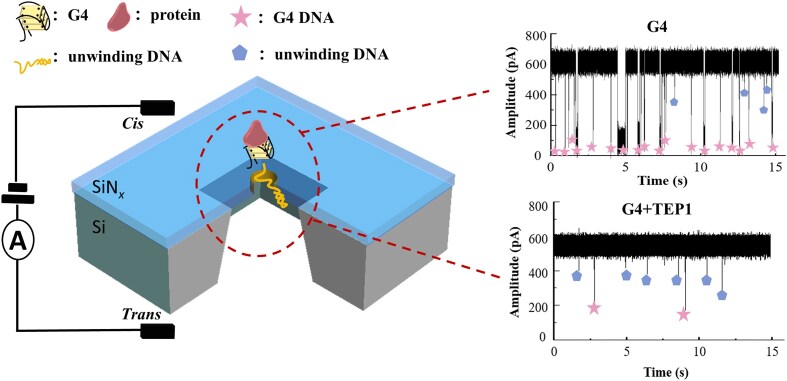

## Introduction

The noncanonical G-quadruplex (G4) structures were proved to be formed under physiologically relevant conditions in guanine-rich sequence in human genome. G4s are inherently long-lived and play regulatory roles in gene expression, replication, recombination, translation, and maintenance of telomere activity [1–4]. Evidence indicates that inappropriate formation of G4s may act as “knots” within genome, which are linked to human diseases and could be unfolded enzymatically [5–7]. The unfolding of stable G4s depends on a suite of nucleic acid helicase proteins.

Helicases are enzymes that are involved in every aspect of DNA metabolism; they use the energy of ATP hydrolysis to walk along and unwind DNA, making them vital for many cellular tasks and the maintenance of genomic integrity [6, 8–11]. Key details of how they work are still not clear, as it is challenging for traditional biophysical techniques to investigate their small (0.6 nm) and fast (∼1 ms) steps [12, 13].

Biochemical analysis of the folding/unfolding of G4 by individual proteins is essential for establishing their activity and specificity for G4s [14]. The ensemble approaches are feasible for the exploration of the stability and interplay of G4s with specific proteins [15–19]. However, to better understand the individual roles that the G4 unwinding protein plays *in vivo*, real-time monitoring of G4 unfolding activities *in vitro* by protein is vital, and single-molecule techniques are uniquely favorable for disclosing the dynamics of G4s with proteins [20, 21]. Traditional techniques such as single-molecule fluorescence resonance energy transfer [22, 23] and magnetic and optical tweezers [24–29] are generally employed for the monitoring of the conformation kinetics of quadruplexes’ unwinding and the interplay dynamics between G4s and proteins. However, the fluorescence labeling or external force manipulation is usually complicated and will change the native features of biomacromolecules.

Nanopore platform is based on label-free single-molecule electrochemical approaches for the capture and characterization of numerous types of targets, including single macromolecules, small molecules, particles, and cells, as well as sensing of metal ions [30–36]. The work principle is recording the translocation ionic current of the analytes under external bias potential across the size-matched nanopore, which is sandwiched in buffer-filled chambers. The single-molecule information, such as the geometry, charge status, and conformation of the threading targets, could be statistically extracted via current fluctuation signals due to physical occupation or interplay with the orifice [37–40]. The applications of nanopore tweezers become a focal point in the manipulation and modulation of the DNA–protein interaction and step control [12, 13, 41, 42]. However, the single-molecule G4 unfolding process by DNA and RNA helicases with nanopore monitoring is not documented.

Herein, we characterized the activity of proteins and proved the existence of folding/unfolding equilibration with a nanopore platform. A tailored nanopore of ∼3.7 nm diameter is utilized for the determination of DNA/RNA-G4 and the unwinding structure after incubation and interaction with proteins. The optimization of the incubation time, concentration of proteins, electrolyte buffer type, and pH conditions result in a maximum unfolding ratio of G4. In addition, DNA unwinding protein is selective to unfold G4 of special topologies such as hybrid G4 rather than parallel G4, while RNA helicase has an effect on all related SARS-CoV sequences. We established a label-free approach for the *in vitro* tracking of the unwinding of nucleic acid quadruplexes by specific proteins and help to understand the interaction rate, activity, and selectivity of the G4 proteins.

## Materials and methods

### Materials

Silicon nitride nano-chips of 3 mm diameter used in this study were ordered from Norcada, Canada. The silicon nitride film of 20 nm thickness was grown on silicon and freestanding on top of a 10-μm^2^ window. The DNA and RNA sequences were purchased from Sangon, Shanghai. Recombinant human telomerase protein component 1 (TEP1) and recombinant human RTEL1 protein (RTEL1) were purchased from Shanghai Abimat. SARS-CoV-2 nsp13 helicase (nsp13) was purchased from Yunxingtai Co., Beijing. ATP was purchased from Xinsheng Baitai Co., Beijing. Tris(hydroxymethyl) amino methane (Tris) was purchased from Shanghai Aldrich Company. Potassium chloride (KCl), cesium chloride (CsCl), lithium chloride (LiCl), magnesium chloride (MgCl_2_), and ethylenediaminetetraacetic acid were purchased from Macklin Company, Shanghai. The translocation buffers were composed of 1 M KCl/1 M CsCl/0.5 M CsCl, 1× TE, pH 5–9; 0.5 M CsCl, 10 mM Tris, 2 mM MgCl_2_, pH 5; 0.5 M CsCl, 10 mM Tris, 5 mM ATP, pH 5; 2 M LiCl, 10 mM Tris, 2 mM MgCl_2_, 5 mM ATP, pH 5–9; and 0.95 M CsCl/0.05 M KCl, 1× TE, pH 7.4. The electrolyte for controlled dielectric breakdown was 1 M KCl and 1× TE (pH 8). All the aforementioned solutions were prepared using HPLC-grade water from a water purification system (Molecular 1850D).

The G-rich DNA/RNA and reference sequences used for nanopore sensing are

C-kit: 5′-CCC GGG C GGG CGCGA GGG A GGGG A GG-3′ (26 nt)

AS1411: 5′-GGT GGT GGT GGT TGT GGT GGT GGT GG-3′ (26 nt)

hTel: 5′-AGGGTTAGGGTTAGGGTTAGGGTT-3′ (24 nt)

T-hTel: 5′-AGGGTTT GGGTTT GGGTTT GGGTT-3′ (24 nt)

T2A-hTel: 5′-AGGGTTATT AGGGTTATT AGGGTTATT AGGGTT-3′ (33 nt)

BCL-2: 5′-GGGC GCGG GAGG AATT GGGC GGG-3′ (23 nt)

Parallel BCL-2: 5′-GGGC GGGA GGAA TTGG GCGG G-3′ (21 nt)

polyA20: 5′-AAAA AAAA AAAA AAAA AAAA-3′ (20 nt)

The G-rich RNA sequences used in this work are

5NH_2_-SAR-CO-ORF1ab-1574 (RNA-1574): 5′-GGUG UUGU UGGA GAAG GUUC CGAA GG-3′ (26 nt)

5NH_2_-SAR-CO-S-24268 (RNA-24268): 5′-GGCU UAUA GGUU UAAU GGUA UUGG-3′ (24 nt)

5NH_2_-SAR-CO-ORF1ab-13385 (RNA-13385): 5′-GGUA UGUG GAAA GGUU AUGG-3′ (20 nt)

5NH_2_-SAR-CO-N-28903 (RNA-28903): 5′-GGCU GGCA AUGG CGG-3′ (15 nt)

### Apparatus

Twenty-five percent of polyacrylamide gel electrophoresis (PAGE) was carried out in 0.5× Tris-Borate-EDTA buffer under a potential of 110 V for 2 h on an apparatus (PowerPac Basic); 2 μM of RNA in 0.2 M KCl or 0.2 M CsCl was melted at 95°C for 5 min before cooling down to room temperature (r.t.) and was left at r.t. for 30 min to form G4 structure before loading to the gel. After Stains-All staining, gels were scanned. Circular dichroism (CD) spectrum was measured on CD spectrometer Chirascan V100 (Applied Photophysics Ltd, Surry, UK), scanning from 192 to 320 nm. The Keithley 2450 in patch clamp testing was controlled by a LabVIEW program to measure the conductivity of SiN*_x_* membranes by current–voltage (*I–V*) curves. The *I–V* record and ionic current blocking of the samples were measured on a patch clamp amplifier (Axopatch 200B), sampled at 100 kHz, low-pass Bessel filtered at 10 kHz, housed in a Faraday cage.

### Nanopore fabrication

SiN*_x_* chips were treated with ethanol and ultrapure water (1:1, v/v), acetone, and oxygen plasma (30 W) to clean the membrane, meanwhile improving hydrophilicity. The chips were then loaded into a flow cell containing 1 M KCl and 1× TE (pH 8) buffer, and an Ag/AgCl electrode was inserted into the chamber and connected to a Keithley 2450. The breakdown process was reported previously [43–45]. It was controlled by precisely controlling the current and the step size, with the breakdown current typically around 400 nA for SiN*_x_* of 20 nm thickness, and the maximum voltage during punching was set to no more than 18 V [46]. The size of the SiN*_x_* nanopore can be calculated by the following equation:


(1) \begin{eqnarray*}
{G} = {\sigma} {\left[ (4L/\pi{d}^{2}) {+} (1/d)\right]}^{-1},
\end{eqnarray*}

where *G* represents the pore conductance that could be measured via the *I–V* test, *σ* refers to the electrical conductivity (11.45 S/m for buffer of 1 M KCl and TE at pH 8), *L* represents the length of the nanopore, equal to the thickness of the SiN*_x_* membrane (20 nm in this case), and *d* represents the pore diameter. The size of the nanopore employed in this work was between 3 and 6 nm (with 3.7 nm for DNA-G4 and their interaction with proteins, and 3–6 nm for the pore size optimization in sensing of RNA-G4).

### Nanopore measurements for DNA/RNA-G4 unfolding by proteins TEP1/RTEL1/nsp13

First, single-stranded G-rich DNA sequences were dissolved in deionized water (RNA sequences in diethylpyrocarbonate water) (DNA sequences: AS1411, hTel, T-hTel, T2A-hTel, BCL-2, parallel BCL-2, C-kit; RNA sequences: RNA-1574, RNA-13385, RNA-24268, RNA-28903) to prepare 100 μM stock solutions, and then they were diluted with test buffer (1 M KCl/1 M CsCl/0.5 M CsCl, 1× TE, pH 5–9); (0.5 M CsCl, Tris, 2 mM MgCl_2_, pH 5); (2 M LiCl, 10 mM Tris, 2 mM MgCl_2_, 5 mM ATP, pH 5–9); or (0.95 M CsCl/0.05 M KCl, 1× TE, pH 7.4), followed by annealing at 95°C for 5 min and cooling down at r.t. for 1 h. This promotes the folding of single-stranded G-rich sequences into the G4 structure. TEP1 was diluted with enzyme-free water into a 3.5 μM stock solution, RTEL1 into a 3.7 μM stock solution, and nsp13 into a 0.5 μM stock solution. They were added to the pre-folded DNA/RNA-G4 solution with distinct molar ratio, and incubated for different times at 37°C to obtain the final samples for the nanopore measurements. DNA/RNA-G4 samples or their mixture with proteins were loaded in the *cis* chamber, and the blank test buffer was added on the *trans* end. Nanopore translocations were recorded before and after incubation of G4 samples with proteins at 100–300 mV.

### Data collection and analysis

Data analysis was performed with Clampfit 11.2 and plotted with Origin. All event spikes were collected at the 80% level of the highest ionic current blockage amplitude. To minimize possible interference from pore size variations, the recorded current blockage amplitude Δ*I* was normalized by dividing by the open pore current *I*_0_. Capture rate is calculated via the number of events recorded from the nanopore translocation in three equal times and taking an average.

Due to the 100 kHz sampling bandwidth limitation and the exclusion of collisions and nanopore/DNA (or RNA) interaction events, only normalized current blockage amplitudes Δ*I/I*_0_ higher than 0.01 were included in the data analysis; all residence time histograms were sketched with a bin size of 0.2 ms and fitted with exponential decay; and normalized current blockage was divided into 40 bins with a Gaussian fitting, except special indication.

## Results and discussion

### hTel-G4 unfolding by TEP1 recorded with a nanopore sensor

**Figure 1.**
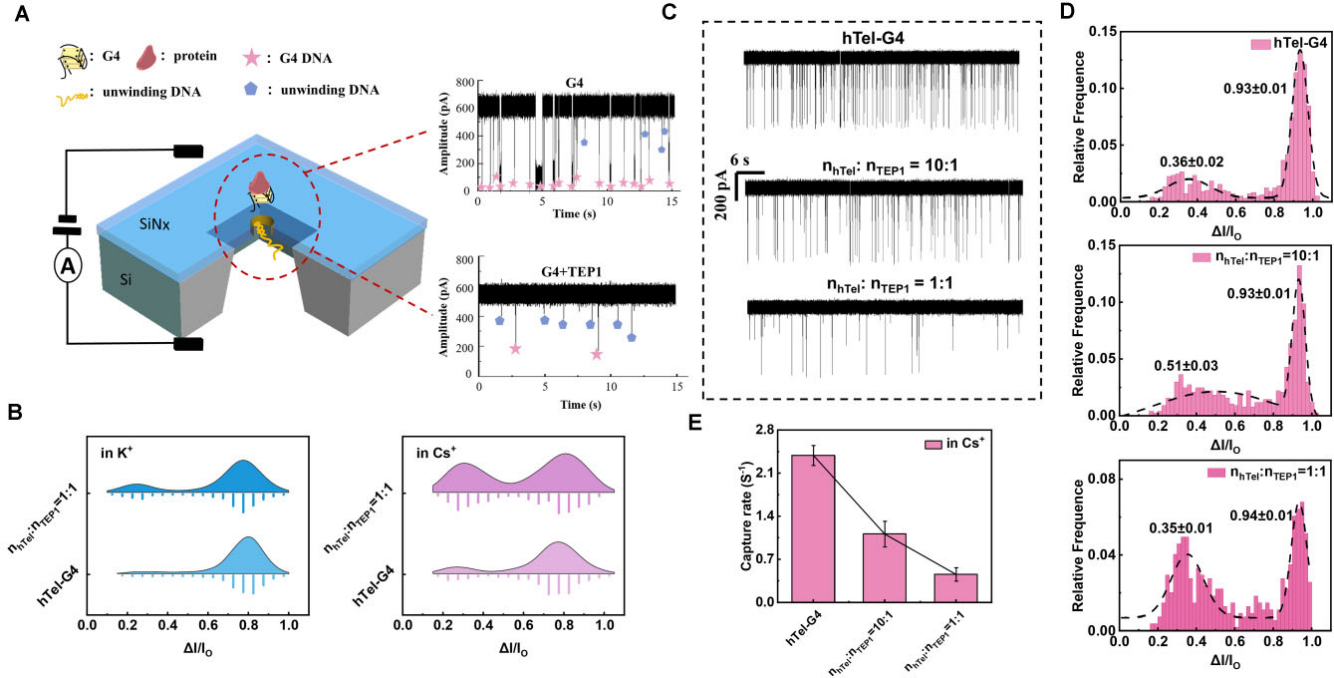
Nanopore determination of quadruplex unfolding by protein TEP1. (**A**) Illustration of the nanopore setup and the raw traces. (**B**) Statistics showing the blockage ratio of individual hTel-G4 and mixed hTel-G4 and TEP1 of equal molar ratio in both 1 M KCl and 1 M CsCl. (**C**) Translocation raw traces in 1 min. (**D**) Histograms of Δ*I/I*_0_. (**E**) Capture rate of individual hTel-G4 and mixed hTel-G4 and TEP1 of distinct molar ratio. All the data were recorded with 10 nM hTel and mixed hTel and TEP1 with different molar ratios for 30 min in 1 M KCl/CsCl and TE (pH 6) in a 3.7 nm nanopore under 100 mV. Error bars represent the standard deviation.

Some vital secondary structures of nucleic acids like G4 are abundant in promotor region and tumor area; their interaction with specific protein discloses important biological cell process and tumor proliferation. Quadruplex structure could be folded *in situ* in the presence of K^+^, Na^+^, and many other mono- or divalent ions to generate stable G4 structure. Their *in situ* unfolding process usually relies on the interaction with specific proteins. In order to discover the interplay condition and modulate the G4 unfolding process, we herein demonstrate a nanopore platform for the G4 unfolding monitoring by both helicase RTEL 1 and protein TEP1 that is associated with telomerase. TEP1 is selected to participate in the unwinding of telomere sequence hTel-G4. The schematic setup of nanopore sensing is exhibited in Fig. 1A. We could see that after the incubation of G4 with TEP1, the translocation events with high blockage amplitude decrease, while the events with low blockade increase, which indicates that the larger G4 was unfolded to single-stranded DNA (ssDNA).

To investigate the unfolding condition and the activation of TEP1 on G4, we screened and searched for the optimized condition. G4 is known to be stable in the presence of K^+^; after incubation of equal mole of TEP1 with pre-folded hTel-G4 for 0.5 h at 37°C in both 1 M KCl and 1 M CsCl, the mixture of hTel-G4 and TEP1 was subjected to nanopore determination and the statistics are exhibited in Fig. 1 and Supplementary Fig. S1. The results reveal that G4 is hard to be unfolded in KCl (Fig. 1B). The increase of the quantity of TEP1 obviously facilitates the unfolding process in 1 M CsCl (Fig. 1C and D), and the overall capture rate decreases due to the conversion of most G4 to the unfolded ssDNA that holds smaller physical dimension (Fig. 1E). Furthermore, the hTel-G4 is instable in 2 M LiCl, exhibiting two distributions in the statistical properties of translocation (Supplementary Fig. S2B); the blockage ratio of 0.52 may be assigned to hTel (fails to form G4 in LiCl) and the *I/I*_0_ of 0.75 is for hTel-G4. Upon addition of TEP1, the proportion of ssDNA (splitting hTel-G4 and incomplete folded hTel in LiCl) increased (Supplementary Fig. S2C), accompanied by a slight decrease in translocation time, which is in accordance with the expectation. We speculated that in 2 M LiCl, TEP1 could interact with hTel-G4, but it is hard to monitor the unwinding process of G4 due to the initial instability of G4 in LiCl.

**Figure 2.**
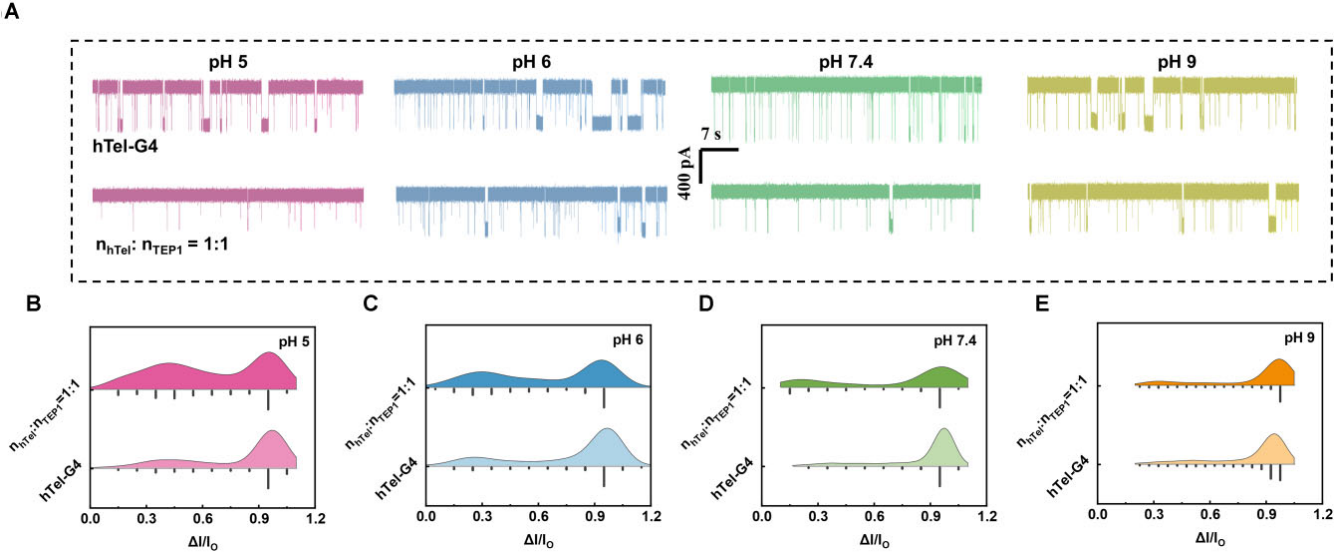
Studies on the enzymatic activity of TEP1 at different pH conditions. (**A**) Translocation raw traces in 1 min. (**B**–**E**) Statistics showing the blockage ratio of individual hTel-G4 and mixed hTel-G4 and TEP1 of equal molar ratio at different pH conditions. All the data were recorded with 10 nM hTel and mixed hTel and TEP1 with equal molar ratio for 30 min in 1 M CsCl and TE at distinct pH in a 3.7 nm nanopore under 100 mV.

pH condition also dominates the interaction status between G4 and protein. The variation of pH from 5 to 9 for the unfolding incubation of 10 nM hTel-G4 with equal mole of TEP1 is examined and the nanopore translocation behavior is displayed in Fig. 2 and Supplementary Figs S3 and S4. From the raw trajectories of nanopore measurements in Fig. 2A, we could find the good capture rate and high signal to noise ratio for the hTel-G4 threading test under all pH conditions in a 3.7-nm nanopore at 100 mV. The incubation of 10 nM TEP1 with 10 nM hTel-G4 in 1 M CsCl for 0.5 h leads to apparent decrease of event frequency in translocation recording. The statistical analysis of the blockage ratio of the incubation mixture in Fig. 2B–E suggests that the unfolded ssDNA with lower blockage ratio is more abundant under weak acidic conditions than under neutral or basic conditions, and the unfolding efficiency and the activity of TEP1 is better under weak acidic conditions.

**Figure 3.**
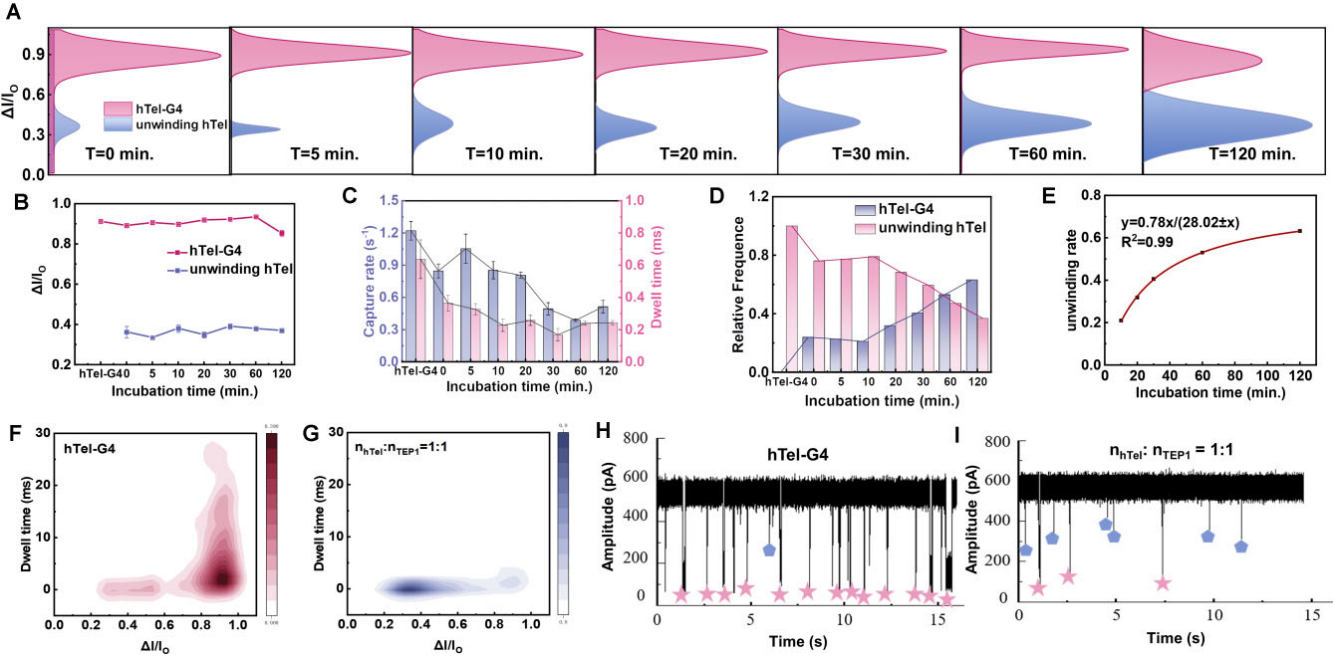
G4 unwinding process recording with nanopore by the variation of incubation time. (**A**) Gaussian fitting of Δ*I/I*_0_. (**B**) Line graphs of Δ*I/I*_0_ as a function of incubation time. (**C**) Histograms of capture rate as a function of incubation time. (**D**) Histograms showing the trend of Δ*I/I*_0_ as a function of incubation time. (**E**) Michaelis–Menten function fitting showing the unwinding rate of hTel-G4 as a function of incubation time. Density graph of the translocation properties for (**F**) hTel-G4 and (**G**) mixed hTel-G4 and TEP1 of equal molar ratio. Original translocation trajectory of (**H**) hTel-G4 and (**I**) mixed hTel-G4 and TEP1 of equal molar ratio. All the data were recorded with 10 nM hTel in 1 M CsCl and TE (pH 5) in a 3.7 nm nanopore at 100 mV. Error bars represent the standard deviation.

**Figure 4.**
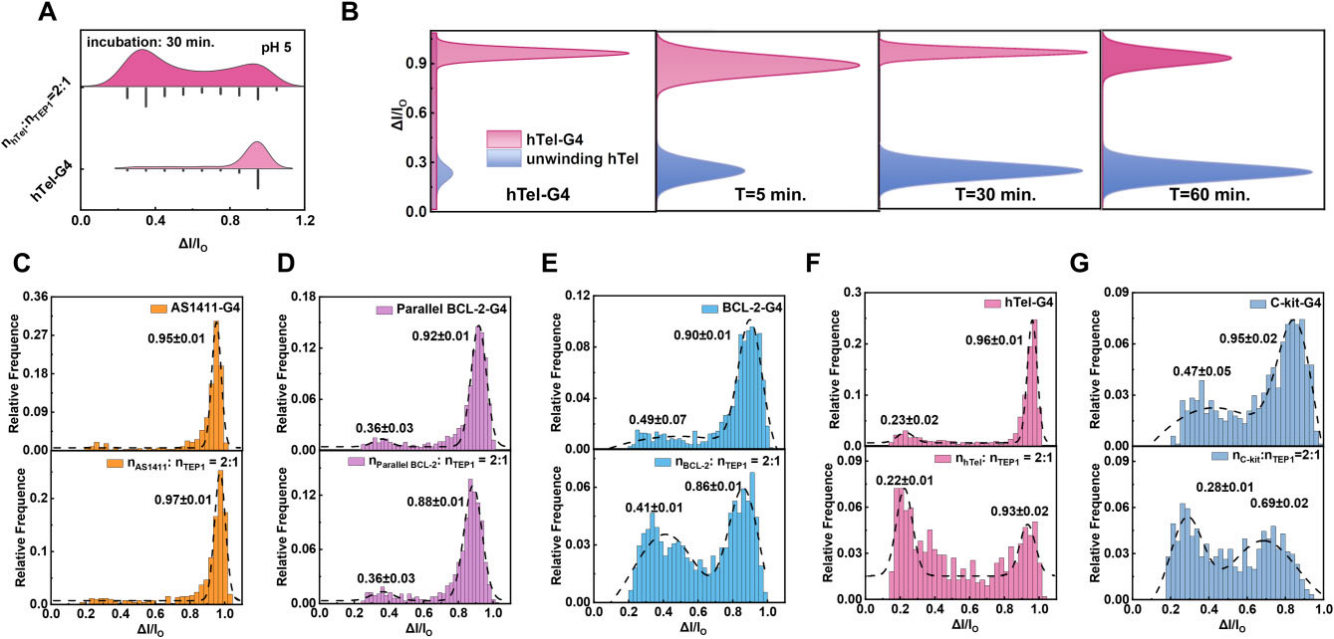
Unfolding selectivity of TEP1 with G4 of distinct topologies. (**A**) Statistics showing the blockage ratio of individual hTel-G4 and mixed hTel-G4 and TEP1 with molar ratio of 2:1 for 30 min. (**B**) Gaussian fitting of Δ*I/I*_0_ for the translocation of individual hTel-G4 and mixed hTel-G4 and TEP1 with molar ratio of 2:1 under different incubation times. Histograms of Δ*I/I*_0_ for the translocation of individual G4 and mixed G4 and TEP1 with molar ratio of 2:1 for 1 h: (**C**) AS1411-G4, (**D**) parallel BCL-2-G4, (**E**) BCL-2-G4, (**F**) hTel-G4, and (**G**) C-kit-G4. All the data were recorded with 20 nM G4 and mixed G4 and TEP1 with molar ratio of 2:1 for 1 h in 0.5 M CsCl and TE (pH 5) in a 3.7 nm nanopore at 150 mV.

Furthermore, we investigate the interaction time to discover the unfolding speed. We conducted the individual assay for each interaction of hTel-G4 and TEP1 with varied incubation duration (0, 5, 10, 20, 30, 60, and 120 min) and subjected the mixture to nanopore measurements. The translocation properties are exhibited in Fig. 3 and Supplementary Figs S5 and S6. Based on the blockage ratio Δ*I/I*_0_ in Fig. 3A and B (the higher value represents the G4 structure, while the lower one refers to unfolded ssDNA), we found that the quantity and capture rate of unfolded ssDNA increase along with the augmentation of incubation time (Fig. 3C and D), and the unfolding increases along with time (Fig. 3E). However, we did not observe the completion of the G4 unfolding after 2-h incubation (Supplementary Fig. S5H and Fig. 3A and G), though the ssDNA dominates the nanopore translocation (Fig. 3A and F–I). We speculate that the folding/unfolding process may be partly reversible in nanopore confined space, and the reversibility is not correlated with protein, as the TEP1 could not translocate through the small nanopore (Supplementary Fig. S7). In addition, to slow down the ssDNA translocation and improve the capture rate, the electrolyte is adjusted from 1 M CsCl to 0.5 M CsCl and the assay was conducted for the incubation mixture of 20 nM hTel-G4 with 10 nM TEP1 at pH 5 for 0.5 h. The statistics of the aperture threading is displayed in Fig. 4A and B. The amount of ssDNA greatly increases, and the disparity between the two fractions’ distribution is apparently notable.

### Unfolding selectivity of TEP1 on DNA-G4

Moreover, we would figure out the selectivity of the unfolding interaction between G4 and TEP1. DNA sequences that could fold into G4 with distinct conformation are selected to conduct the assays (BCL-2, para-BCL-2, AS1411, C-kit, hTel; the sequence details are displayed in the “Materials and methods” section) and the statistics of the translocation properties are demonstrated in Fig. 4 and Supplementary Fig. S8. It is proved that the incubation of 20 nM hTel with 10 nM TEP1 for 1 h in 0.5 M CsCl at pH 5 under 150 mV could lead to efficient unfolding (Fig. 4A and B), and unfolding started just after 5-min incubation and does not change significantly after 0.5 h of incubation (Fig. 4B). The same incubation condition is used for other G4 sequences, and the statistical results are displayed in Fig. 4C–G. Based on the blockage ratio of the incubation mixture, we recorded significant amount of the unfolding ssDNA from BCL-2 and hTel, while almost no ssDNA is captured for para-BCL-2 and AS1411, indicating the selective unfolding kinetics of TEP1 with G4 that holds hybrid structures. As for the sequence C-kit, the G4 formation is not completed, and the incubation with TEP1 promotes significant unfolding as shown in the change of histogram distribution (Fig. 4G).

### Comparison of the unwinding activity of RTEL1 with TEP1 on hTel

We compare the interaction of G4 with TEP1 that is associated with telomerase and helicase RTEL1, a known helicase for G4 unwinding in gene promotor region to maintain the integrity of telomere [47]. We select hTel as a basic sequence, and vary the base type and length in loop in order to verify the influence of loop on the G4 stability. These sequences (hTel, T2A-hTel, and T-hTel) were pre-folded into G4 conformations under annealing conditions (in 1 M KCl, 1× TE, pH 7.4, at 95°C for 5 min, then cooling down to r.t.), and we systematically characterized structural disparity by the resistive current blockade signals through nanopore translocation. The molar ratio of RTEL1:G4 was set at 1:2 (the mixture was incubated for 1 h at 37°C and cooled down before subjecting to nanopore recording) as that for TEP1 to validate the activity of helicase RTEL1 in unwinding G4 structure in 1 M KCl.

In a single 3.7-nm nanopore, the three pre-folded G4s (hTel, T2A-hTel, and T-hTel) and their incubated mixture (G4 and RTEL1) were separately loaded in the chamber. Upon the interaction of RTEL1, the distribution of current blockage ratio Δ*I/I*_0_ values for the three sequences showed nearly no change compared with the individual hTel-G4 (Supplementary Fig. S9). This observation is likely attributed to the high structural stability of G4s formed in KCl; little part of splitting ssDNA may not significantly alter the overall Δ*I/I*_0_ distribution pattern. However, a reduction in dwell time was observed (Supplementary Fig. S9D), suggesting a possible existence of partial unwinding of G4 to ssDNA by RTEL1 in KCl. In this assay, the sharp narrow Gaussian distribution of Δ*I/I*_0_ at 0.94, 0.93, and 0.97 for the three sequences illustrates a nearly complete formation of stable G4 with a relatively single translocation motion (Supplementary Fig. S9); therefore, no significant influence of loop (length and base type) on the stability of G4 folded from hTel was found, and it may also depend on the sequences.

To investigate whether the optimized conditions of TEP1 are applicable to RTEL1, we conducted assays in 0.5 M CsCl instead of 1 M KCl at pH 5 with a 1-h incubation, using the same nanopore in size. As shown in Supplementary Fig. S10, the addition of RTEL1 in G4 in 0.5 M CsCl resulted in a marked increase in the proportion of splitting ssDNA peaks (Supplementary Fig. S10), indicating a significant unfolding activity of RTEL1 on hTel-G4. For a reference of ssDNA, we performed the control assay with homopolymer polyA20, and the translocation properties are apparently distinct with G4 (Supplementary Fig. S11).

**Figure 5.**
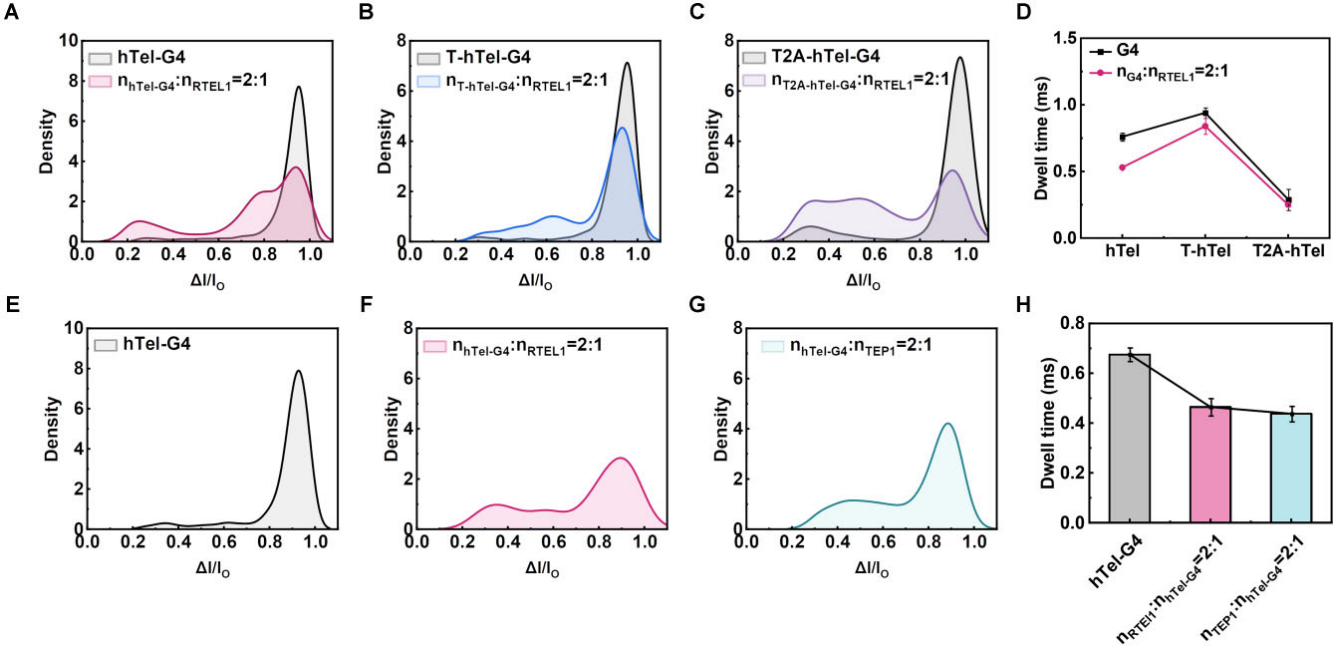
Comparison of the unwinding activity of RTEL1 with TEP1 on hTel-G4 via nanopore characterization. Overlapped distribution of Δ*I/I*_0_ (**A**–**C**); overlapped line graphs of dwell time (**D**) of individual hTel-G4 and mixed G4 and RTEL1 with molar ratio of 2:1. Gaussian distribution of Δ*I/I*_0_ of (**E**) hTel-G4, (**F**) mixed G4 and RTEL1 with molar ratio of 2:1, and (**G**) mixed G4 and TEP1 with molar ratio of 2:1. (**H**) Histograms of dwell time of individual G4 and mixed G4 and RTEL1/TEP1 with molar ratio of 2:1. All the data were recorded with 20 nM hTel and mixed hTel and RTEL1/TEP1 with molar ratio of 2:1 for 1 h in 0.5 M CsCl, Tris and 5 mM ATP (pH 5) in a 3.7 nm nanopore under 150 mV. Error bars represent the standard deviation.

Finally, we compared the interaction of RTEL1 and TEP1 with hTel-G4 under optimized conditions. The statistics of nanopore translocation are presented in Fig. 5 and Supplementary Fig. S12. From the overlapped Gaussian distribution of Δ*I/I*_0_ in Fig. 5A–C, the splitting disparity is evident for the three sequences in the presence of helicase RTEL1, and a reduction of blockage duration is also observed (Fig. 5D). However, the incubation and interaction of hTel with both RTEL1 and TEP1 result in similar proportions of splitting ssDNA (Fig. 5F and G), and the blockage duration is close for both cases (Fig. 5H), indicating also a significant interaction activity of both RTEL1 and TEP1 with hTel-G4.

### Discrimination of distinct SARS-CoV RNA-G4 and the interaction with nsp13 via nanopore readouts

SARS-CoV-2 is a β-coronavirus with a positive-sense single-stranded RNA genome that is 29.9 kb long. Among its viral-encoded proteins, the nsp13 helicase plays a crucial role as a regulator of G4 structures. The pyramidal nsp13 consists of two typical SF1 helicase RecA domains, an N-terminal zinc-binding domain (ZBD), a stem, and a coronavirus-specific 1B domain. The ZBD domain is essential for supporting the helicase activity of nsp13 [48–50].

Herein, some typical G-rich SARS-CoV gene sequences (RNA-1574, RNA-13385, RNA-24268, and RNA-28903) with distinct sequence in length are selected for the conformation identification and further unfolding investigation. We first optically characterized the size of the four RNA-G4s by both UV–Vis and CD for the four RNA G-rich SARS-CoV-2 sequences in 0.1 M KCl (Supplementary Fig. S13). The UV–Vis spectra indicate that all four RNA-G4s exhibited strong absorption at ∼260 nm without significant distinction (Supplementary Fig. S13A). CD spectroscopy demonstrates characteristic positive signals at 220 and 260 nm, indicating that all sequences adopt parallel G4 conformation (Supplementary Fig. S13B). PAGE characterization was also conducted with 2 μM samples in both 0.2 M KCl and 0.2 M CsCl, and the results are presented in Supplementary Fig. S14. The bands are similar in both electrolyte media, with a size order of RNA-1574-G4 > RNA-24268-G4 > RNA-13385-G4 > RNA-28903-G4 that corresponds to the order of the sequence length, and all the RNA-G4s tend to form dimers as compared with the marker size, except RNA-28903-G4 [51]; however, they performed differently via nanopore recording. The four pre-folded RNA-G4 sequences in 1 M KCl and TE (pH 7.4) were subjected to nanopore threading measurements. The statistics of the translocation behavior with nanopores of different diameters (3.8–5.2 nm) is demonstrated in Supplementary Fig. S15. The four RNA-G4s could be generally differentiated. With a nanopore <4.4 nm, the RNA-28903-G4 was clogged (Supplementary Fig. S15A), and the Δ*I/I*_0_ values of RNA-1574, RNA-13385, and RNA-24268 were close to each other (Supplementary Fig. S15B and C). With a larger pore of 4.9–5.2 nm, the translocation of RNA-28903-G4 could be recorded and the ΔI*/I*_0_ of the four RNA-G4s is distinctive (Supplementary Fig. S15D–F). In an appropriate pore of 4.2 nm, the blockage ratio and dwell time of the three RNA-G4s in KCl are identifiable (Supplementary Fig. S15G and H). However, it is difficult to discriminate the three RNA-G4s with the assays under the same conditions in LiCl (Supplementary Fig. S16). We deduced that the G4 conformation is distinct in confined nanopore from the gel mesopore.

**Figure 6.**
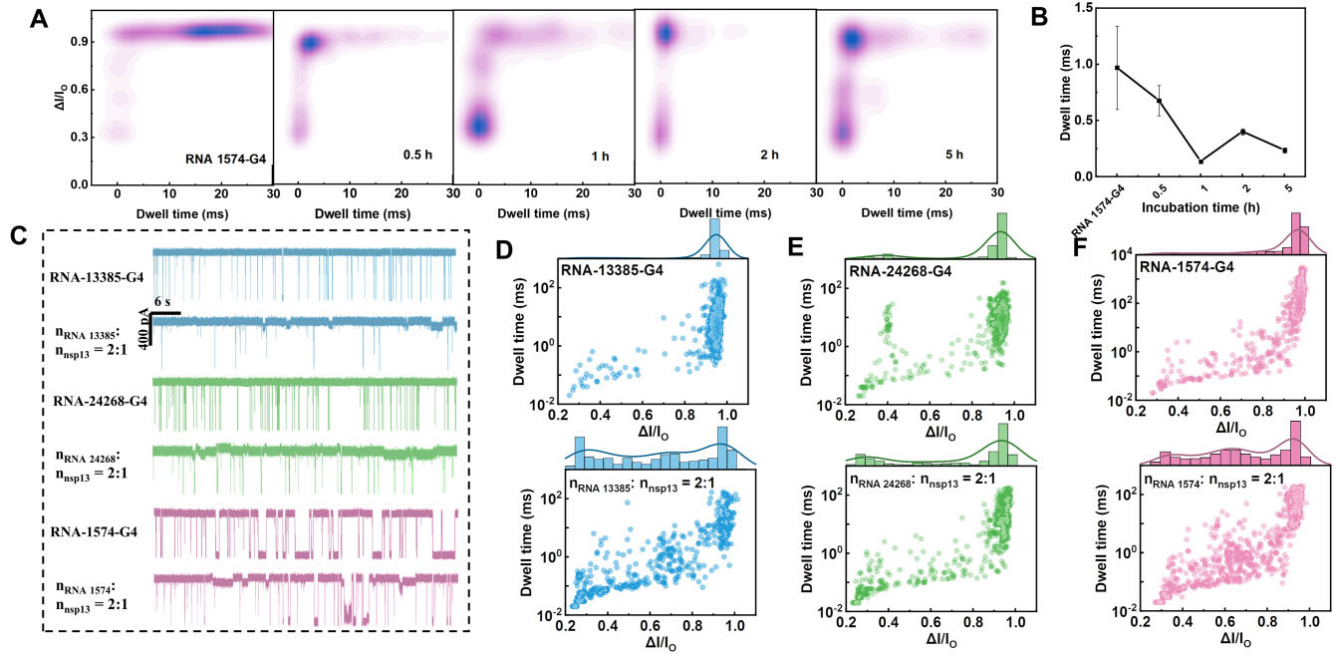
Nanopore translocation properties showing the unfolding of RNA-G4 with nsp13 helicase. (**A**) Density graphs showing the translocation properties of individual RNA-1574-G4 and mixed RNA-1574-G4 and nsp13 with molar ratio of 10:1 under distinct incubation time. (**B**) Line graph of the blockage duration for the samples in panel (A) as a function of incubation time. (**C**) Translocation raw traces for the indicated samples in 1 min. Scatter plots and histograms showing the translocation behaviors for the samples. (**D**) Individual RNA-13385-G4 and mixed RNA-13385-G4 and nsp13 with molar ratio of 2:1, (**E**) individual RNA-24268-G4 and mixed RNA-24268-G4 and nsp13 with molar ratio of 2:1, and (**F**) individual RNA-1574-G4 and mixed RNA-1574-G4 and nsp13 with molar ratio of 2:1. All the data were recorded with 10 nM RNA-G4 and mixed RNA-G4 and nsp13 with molar ratio of 2:1 for 1 h in 2 M LiCl, Tris, 2 mM MgCl_2_ and 5 mM ATP (pH 5) in a 3.6-nm nanopore at 100 mV. Error bars represent the standard deviation.

Furthermore, we selected RNA-1574, which holds the longest chain in length and the largest G4 in dimension, for unfolding modulation with RNA helicase nsp13. The incubation of 10 nM RNA-1574-G4 with 5 nM nsp13 for 30 min results in apparent unfolding of G4 to ssDNA that holds lower Δ*I/I*_0_ and short blockage duration (Supplementary Fig. S17), recorded in a 3.6-nm nanopore in 0.95 M CsCl/0.05 M KCl and TE (pH 7.4). In order to improve the activity of helicase nsp13 and accelerate the G4 unfolding speed, an optimized 2 M LiCl system (Supplementary Fig. S18) was selected and 2 mM MgCl_2_ and 5 mM ATP were added [52]. Likewise, the better unfolding efficiency of helicase is observed under weak acidic conditions (Supplementary Fig. S18E–H). The increase of the incubation time to 2–5 h does not promote the completion of the unfolding process, leading to the reversibility to G4 (Fig. 6A and Supplementary Fig. S19). We deduced that the activity of the helicase nsp13 is limited in 1 h, and it undergoes degradation with time. From the raw translocation traces in Fig. 6C, we could see the decrease of the capture frequency for all the RNA-G4s after incubation with 50% molar ratio of helicase nsp13 for 1 h. The statistical scatter plots of the three individual RNA-G4s show a relatively concentrative distribution of the translocation trajectories with a Δ*I/I*_0_ value at ∼0.95, with little dispersive events of ssDNA (Fig. 6D–F). The incubation of RNA-G4 with 50% of helicase nsp13 for 1 h provokes apparent unfolding, and intermediate and ssDNA generation with a broad distribution and lower Δ*I/I*_0_ (Fig. 6D and F). The dwell time of the samples significantly decreased after incubation of the RNA-G4 with helicase nsp13 (Supplementary Fig. S20), indicating the abundant translocation of unfolded ssDNA. It is worth mentioning that our SiN*_x_* nanopore system generally performs the target sensing with high concentration of electrolyte (0.5–1 M) to guarantee the sufficient signal to noise ratio, though low concentration of buffer solution also works for the detection of proteins, DNA, and their complex via interaction [44, 53, 54].

## Conclusion

To sum up, we provided a label-free single-molecule nanopore platform for the monitoring and regulation of G4 interaction with proteins. Twenty nanomolar of hTel-G4 could be unfolded by 10 nM of both TEP1 that is associated with telomerase and helicase RTEL1 in 1 h under weak acidic conditions in 0.5 M CsCl, and unwinding ssDNA is recorded via nanopore translocation in a size-matched nanopore of 3.7 nm diameter under 150 mV. However, the increase of the incubation time does not promote the completion of the unfolding process, and TEP1 selectively interplays with DNA-G4 that holds hybrid topology. G-rich RNA sequences originated from SARS-CoV are selected for conformation discrimination and unfolding modulation with RNA helicase nsp13. The four RNA-G4s that hold similar sequences in length are distinctive via PAGE and nanopore characterization; the size order is contradictive in the two approaches due to confined nanospace and mesopore of gel. Ten percent of the nsp13 is efficient for RNA-1574-G4 unfolding in 1 h in the presence of Mg^2+^ and ATP. The prolongation of the incubation duration to 5 h results in reversibility in folding/unfolding, and the increase of the helicase ratio to 50% leads to the unfolding of all the three RNA-G4s without apparent selectivity. This work demonstrates an *in vitro* manner for monitoring the unfolding process of quadruplex DNA/RNA and proteins, which will be insightful for understanding the interplay of the nucleic acids with enzyme and the related biological process and mechanism.

## Supplementary Material

gkaf547_Supplemental_File

## Data Availability

The data that support the findings of this study are available from the corresponding authors on request.
