# Supplementary material for: Unwinding process of DNA/RNA quadruplexes by proteins under label-free nanopore monitoring
Source: Nucleic Acids Res. 2025 Jun 26;53(12):gkaf547. doi: 10.1093/nar/gkaf547 (PMC12199145; doi:10.1093/nar/gkaf547)
Supplement: gkaf547_Supplemental_File [file gkaf547_supplemental_file.pdf]

Supplementary Information for:

Unwinding Process of DNA/RNA Quadruplexes by Proteins under Label-Free Nanopore Monitoring

Meili Ren[1,2,5#], Ting Weng[1#] , Liyuan Liang[1*], Xun Chen[1], Daixin Liu[1], Shaoxi Fang[1], Rong Tian[1], Wanyi Xie[1], Liang Wang[1], Deqiang Wang[1], Chunyu Zeng[1,3,4,5*]

[1]Chongqing Key Laboratory of Multi-scale Manufacturing Technology, Chongqing Institute of Green and Intelligent Technology & Chongqing School, University of Chinese Academy of Science, Chongqing 400714, P. R. China

[2]Chongqing Jiaotong University, Chongqing 400014, P. R. China

[3]Department of Cardiology, Daping Hospital, Third Military Medical University, Chongqing, PR China

[4]Key Laboratory of Geriatric Cardiovascular and Cerebrovascular Disease Research, Ministry of Education of China, Chongqing Key Laboratory for Hypertension Research, Cardiovascular Clinical Research Center, Chongqing Institute of Cardiology, Chongqing, PR China

[5]Department of Cardiology, The First Affiliated Hospital of Qunming Medical University, Qunming, Yunnan Province, PR China

The authors wish it to be known that, in their opinion, the first two authors should be regarded as joint First Authors.

*Corresponding Authors' e-mail: liangliyuan@cigit.ac.cn, chunyuzeng01@163.com

Fig. S1. Nanopore translocation properties of G4 and mixed G4 and TEP1 helicase in distinct electrolyte conditions........3
Fig. S2. Nanopore translocation properties of G4 and mixed G4 and TEP1 helicase in 2 M LiCl 4
Fig. S3. Nanopore translocation statistics of G4 and mixed G4 and TEP1 helicase at different pH conditions. 5
Fig. S4. Nanopore translocation statistics of G4 and mixed G4 and TEP1 helicase at different pH conditions........6
Fig. S5. G4 unwinding process recording with nanopore under distinct incubation time........7
Fig. S6. G4 unwinding process recording with nanopore under distinct incubation time........8
Fig. S7. Nanopore determination of TEP1 and nsp13 helicase........9
Fig. S8. Unfolding selectivity of TEP1 with G4 of distinct topologies........10
Fig. S9. Nanopore translocation properties of G4 and mixed G4 and RTEL1 helicase in 1 M KCl........11
Fig. S10. Nanopore translocation properties of G4 and mixed G4 and RTEL1 helicase in 0.5 M CsCl........12
Fig. S11. Comparison of nanopore detection between single-stranded DNA with similar base numbers and G4 structures........13
Fig. S12. Nanopore-based comparison of hTel-G4 unwinding by RTEL1 and TEP1 helicases........14
Fig. S13. Ultraviolet-visible (UV-Vis) absorption spectra and circular dichroism (CD) spectra of four RNA-G4s........15
Fig. S14. PAGE characterization........16
Fig. S15. Identification of different RNA-G4 sequences with nanopores........17
Fig. S16. Identification of different RNA G4 sequences with nanopores in 2 M LiCl........18
Fig. S17. Nanopore translocation properties of G4 and mixed G4 and nsp13 helicase with distinct nsp13 molar ratio. 19
Fig. S18. Nanopore translocation properties of G4 and mixed G4 and nsp13 helicase in distinct electrolyte conditions and pH conditions........20
Fig. S19. RNA 1574-G4 unwinding process recording with nanopore under distinct incubation time........21
Fig. S20. Unfolding selectivity of nsp13 with G4 of distinct topologies........22

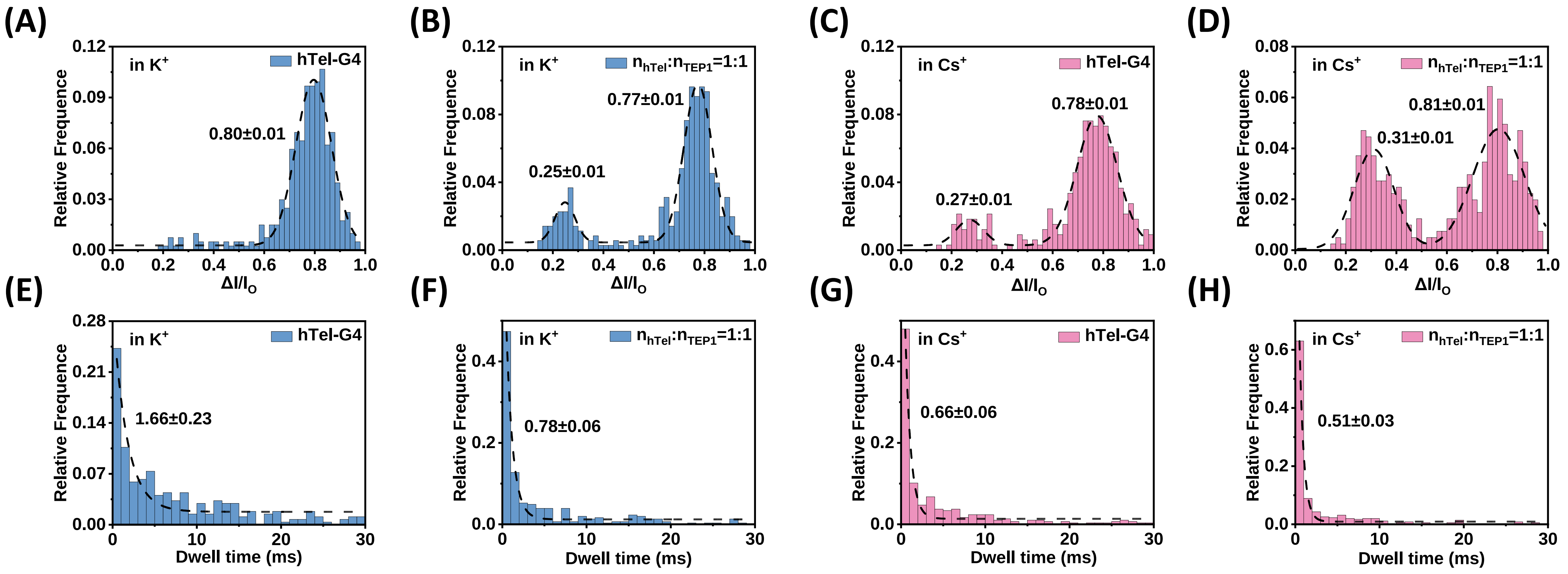

S1. Nanopore translocation properties of G4 and mixed G4 and protein TEP1 in distinct electrolyte conditions. Histograms of (A-D) $\Delta I/I_o$, (E-H) dwell time of individual hTel-G4 and mixed hTel-G4 and TEP1 of equal molar ratio in both 1 M KCl and 1 M CsCl. All the data were recorded with 10 nM hTel and mixed hTel and TEP1 with equal molar ratio for 30 min. in 1 M KCl/ CsCl, TE, pH 6 in a 3.7 nm nanopore under 100 mV.

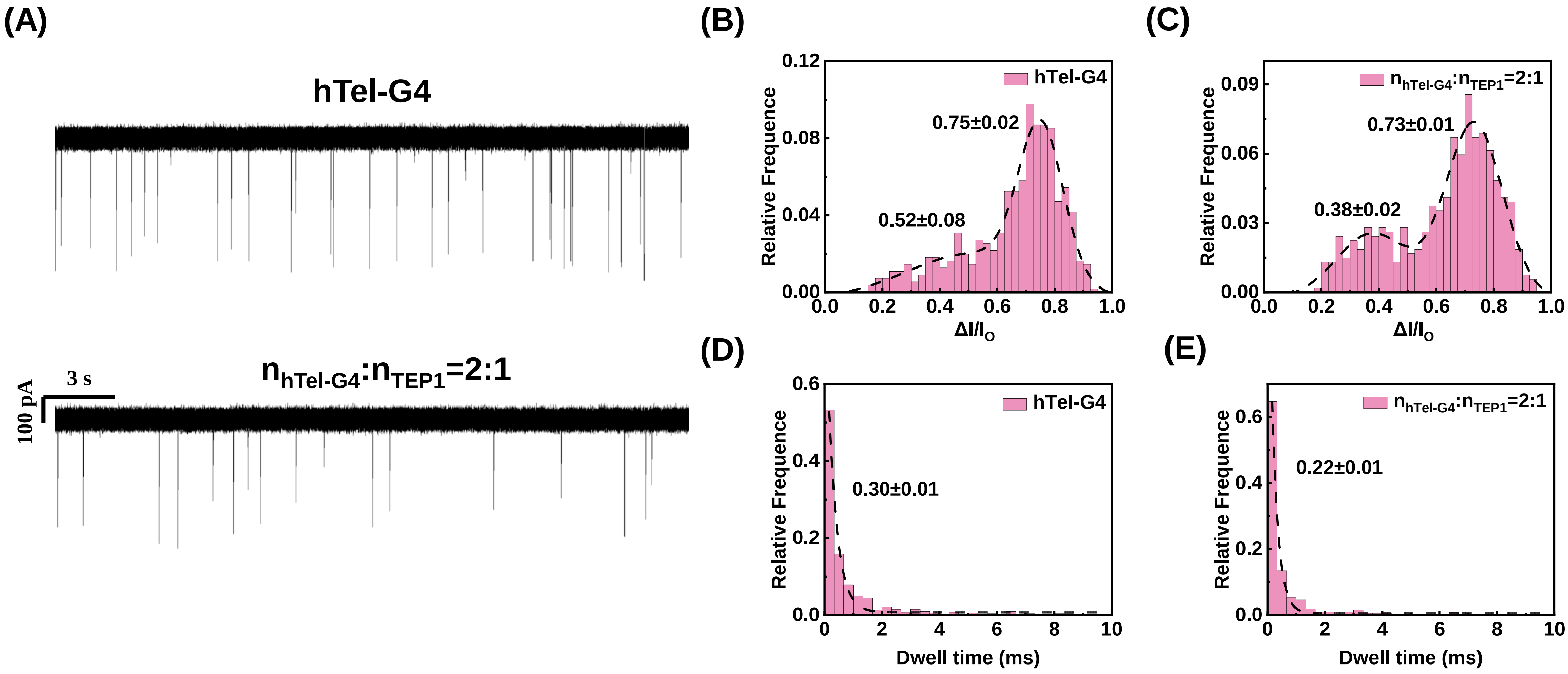

S2. Nanopore translocation properties of G4 and mixed G4 and protein TEP1 in 2 M LiCl. (A) Translocation raw traces in 30 s. Histograms of (B-C) $\Delta I/I_o$, (D-E) dwell time of individual hTel-G4 and mixed hTel-G4 and TEP1 with molar ratio of 2:1 in 2 M LiCl. All the data were recorded with 20 nM hTel and mixed hTel and TEP1 with molar ratio of 2:1 for 30 min. in 2 M LiCl, TE, pH 5 in a 3.7 nm nanopore under 100 mV.

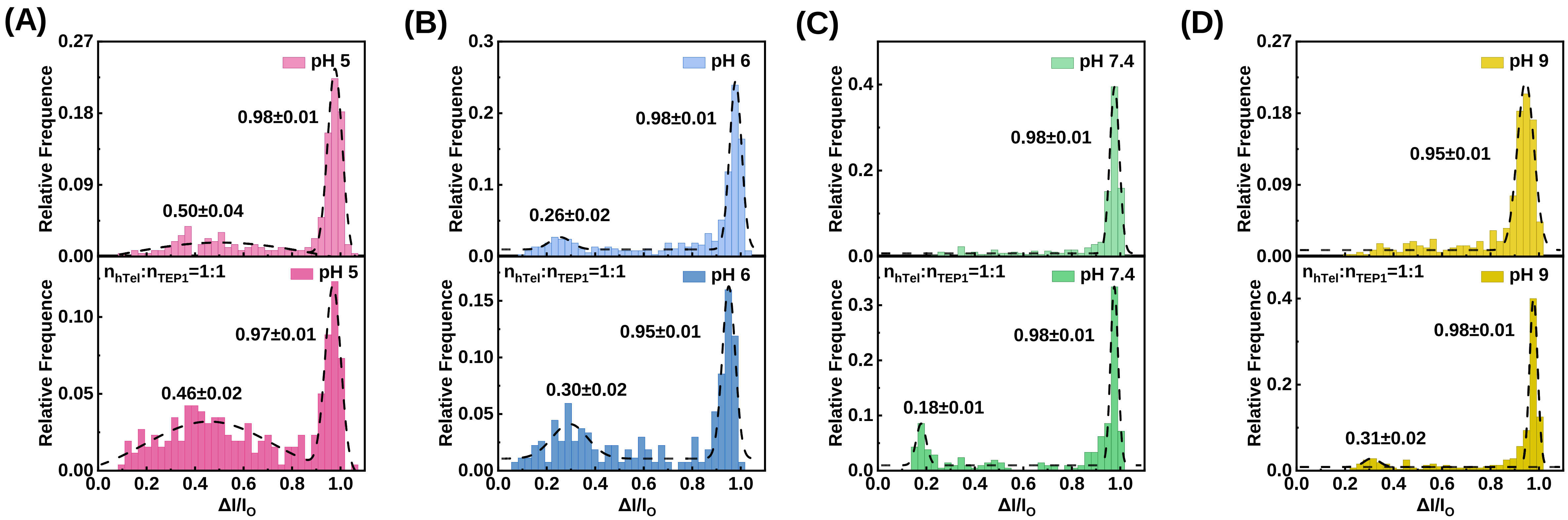

S3. Nanopore translocation statistics of G4 and mixed G4 and protein TEP1 at different pH conditions. (A-D) Histograms of $\Delta I/I_o$ of individual hTel-G4 and mixed hTel-G4 and TEP1 of equal molar ratio at different pH conditions. All the data were recorded with 10 nM hTel and mixed hTel and TEP1 with equal molar ratio for 30 min. in 1 M CsCl, TE, in a 3.7 nm nanopore under 100 mV.

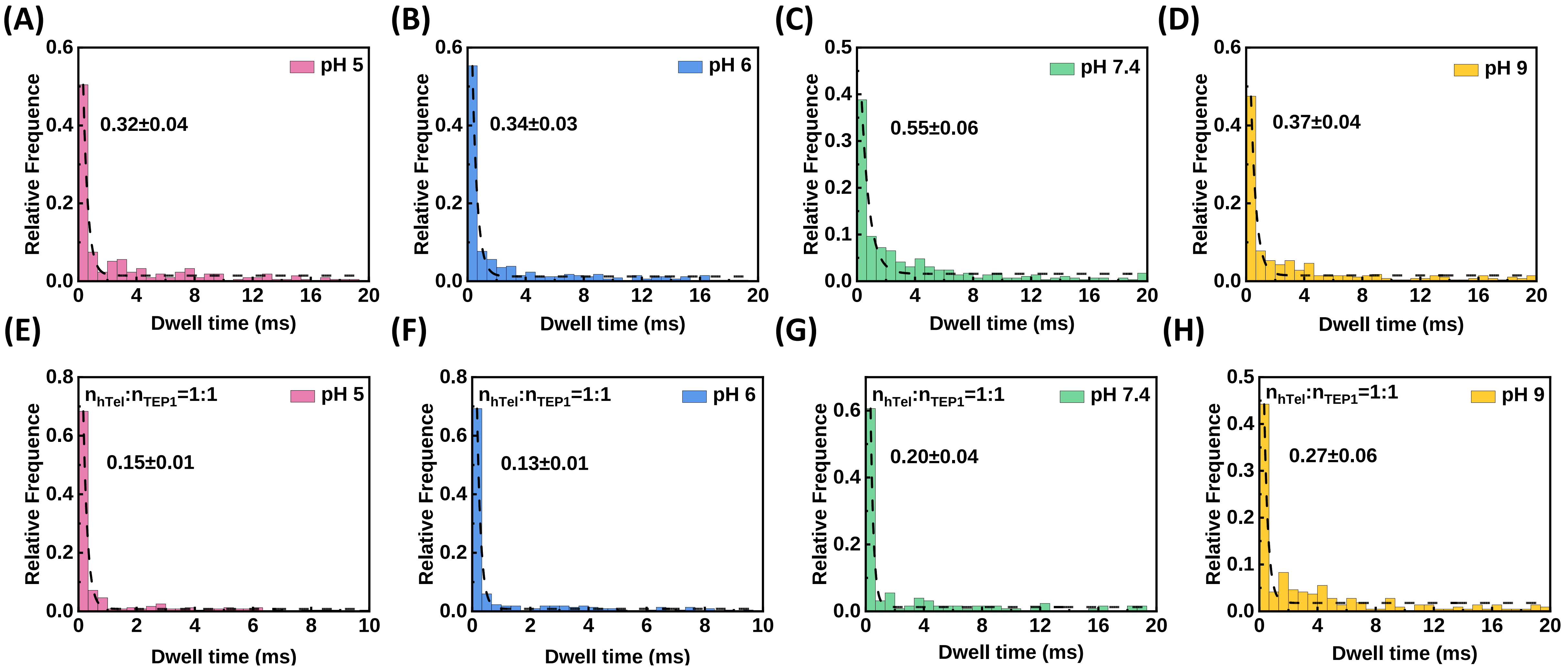

S4. Nanopore translocation statistics of G4 and mixed G4 and protein TEP1 at different pH conditions. Histograms of dwell time of (A-D) individual hTel-G4 and (E-H) mixed hTel-G4 and TEP1 of equal molar ratio at different pH conditions. All the data were recorded with 10 nM hTel and mixed hTel and TEP1 with equal molar ratio for 30 min. in 1 M CsCl, TE, in a 3.7 nm nanopore under 100 mV.

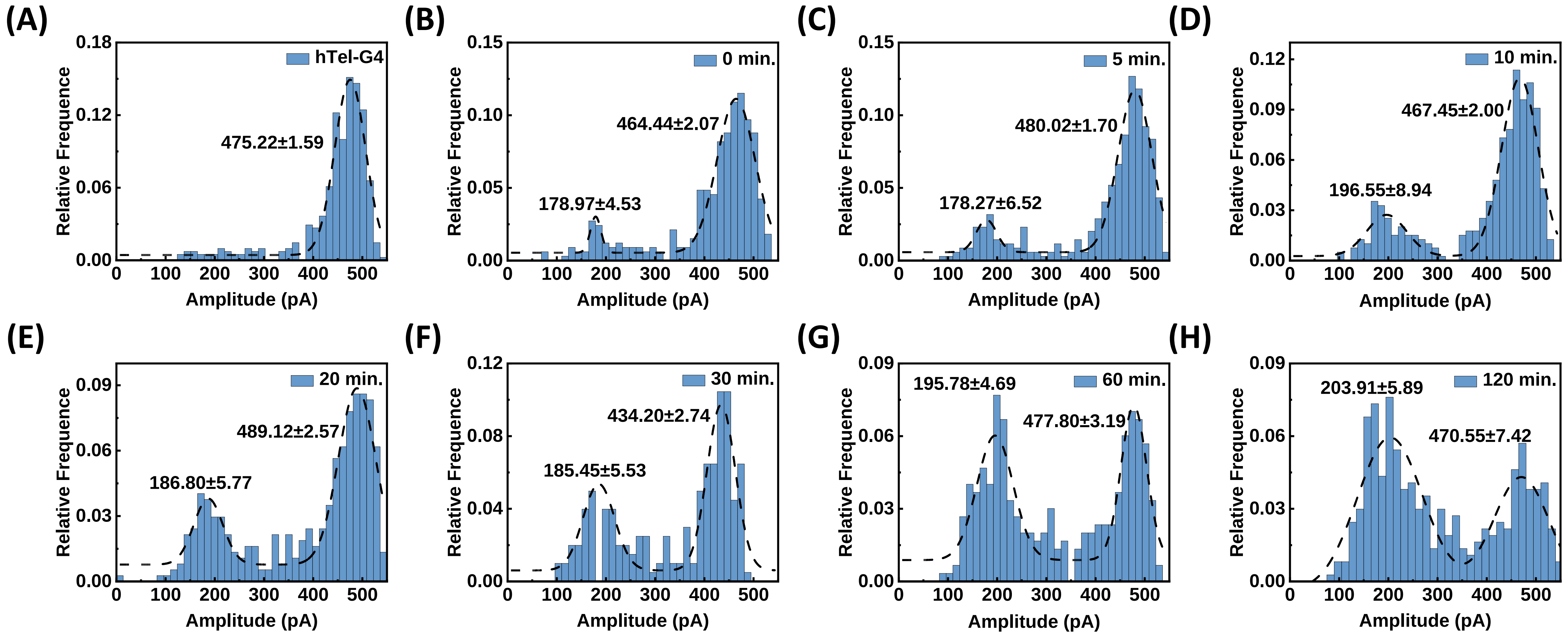

S5. G4 unwinding process recording with nanopore under distinct incubation time. Histograms of blockage amplitude of (A) individual hTel-G4 and (B-H) mixed hTel-G4 and TEP1 of equal molar ratio under different incubation time. All the data were recorded with 10 nM hTel and mixed hTel and TEP1 with equal molar ratio in 1 M CsCl, TE in a 3.7 nm nanopore under 100 mV.

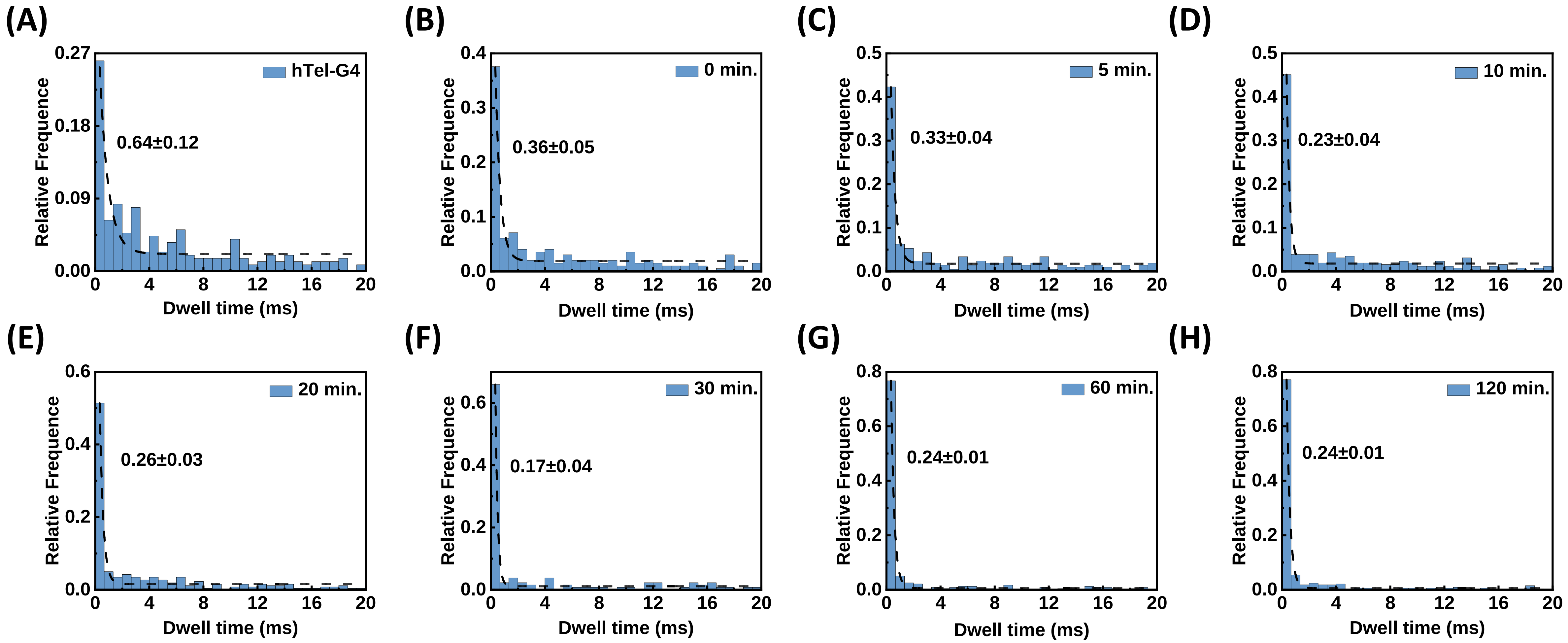

S6. G4 unwinding process recording with nanopore under distinct incubation time. Histograms of dwell time of (A) individual hTel-G4 and (B-H) mixed hTel-G4 and TEP1 of equal molar ratio under different incubation time. All the data were recorded with 10 nM hTel and mixed hTel and TEP1 with equal molar ratio in 1 M CsCl, TE in a 3.7 nm nanopore under 100 mV.

(A)

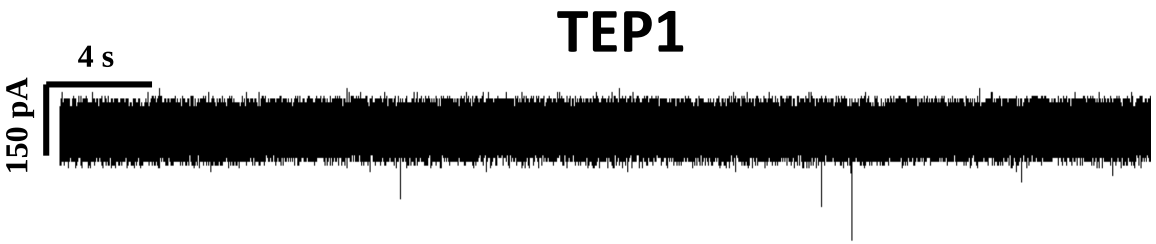

(B)

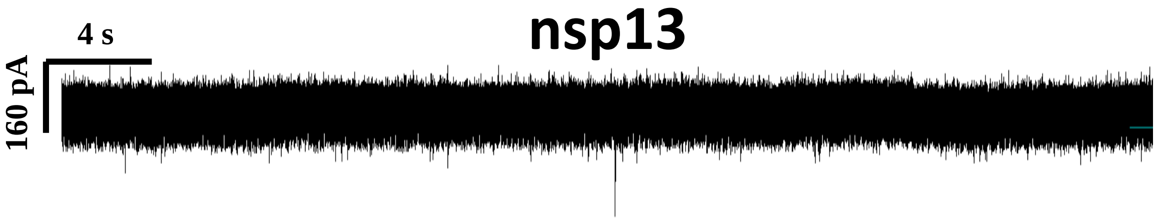

S7. Nanopore determination of protein TEP1 and nsp13 helicase. Translocation raw traces in 1 min. for (A) TEP1 and (B) nsp13. protein TEP1 translocation raw traces was recorded with 20 nM TEP1 in 1 M CsCl, TE, pH 5 in a 3.7 nm nanopore at 100 mV, nsp13 helicase translocation raw traces was recorded with 10 nM nsp13 in 2 M LiCl, 2 mM $MgCl_2$, 5 mM ATP, pH 5 in a 3.7 nm nanopore under 100 mV.

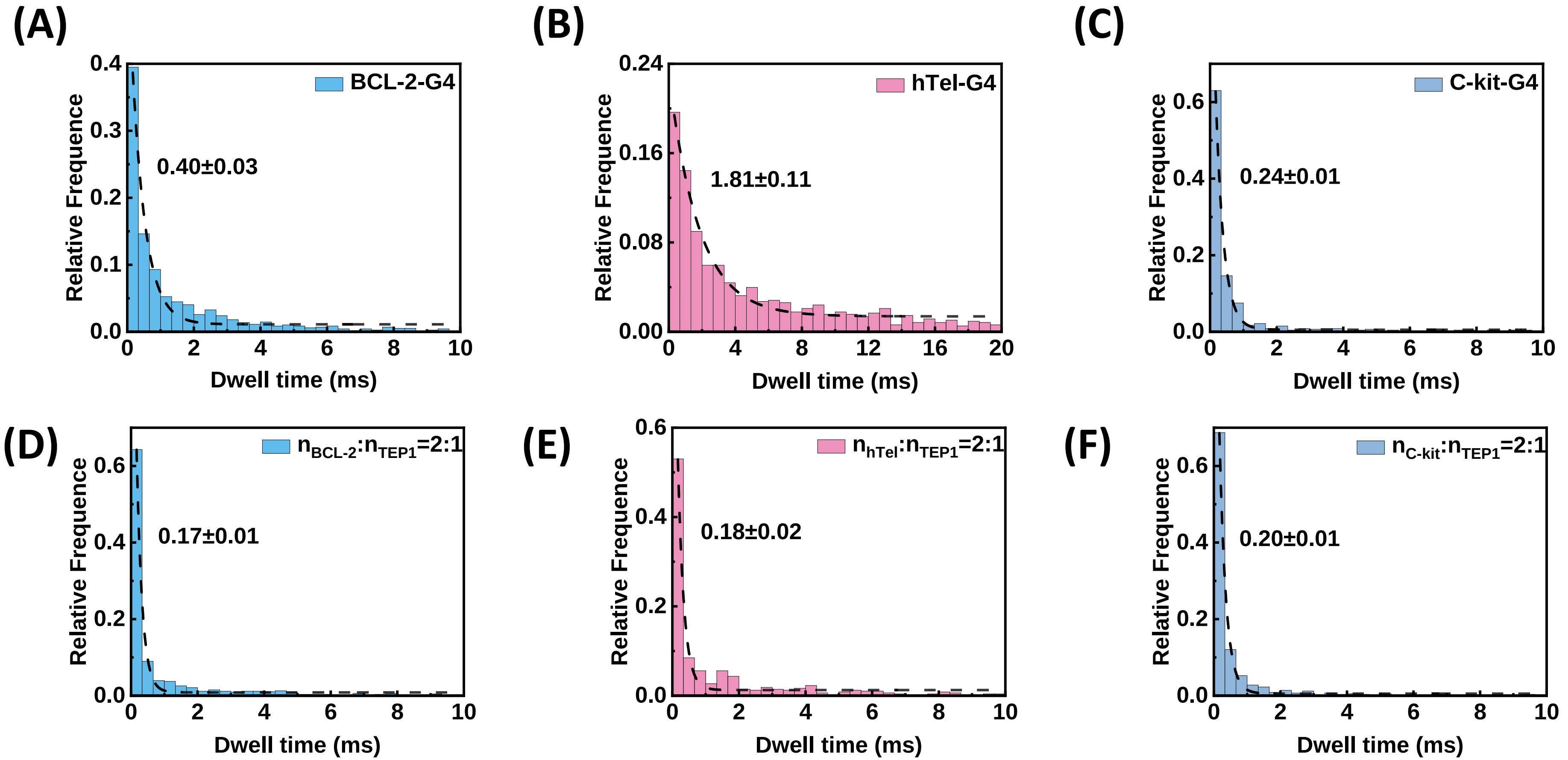

S8. Unfolding selectivity of TEP1 with G4 of distinct topologies. Histograms of blockage duration of the translocation of (A-C) individual G4 and (D-F) mixed G4 and TEP1 with molar ratio of 2:1 for 1 h. All the data were recorded with 20 nM G4 and mixed G4 and TEP1 with molar ratio of 2:1 for 1 h in 0.5 M CsCl, TE, pH 5 in a 3.7 nm nanopore at 150 mV.

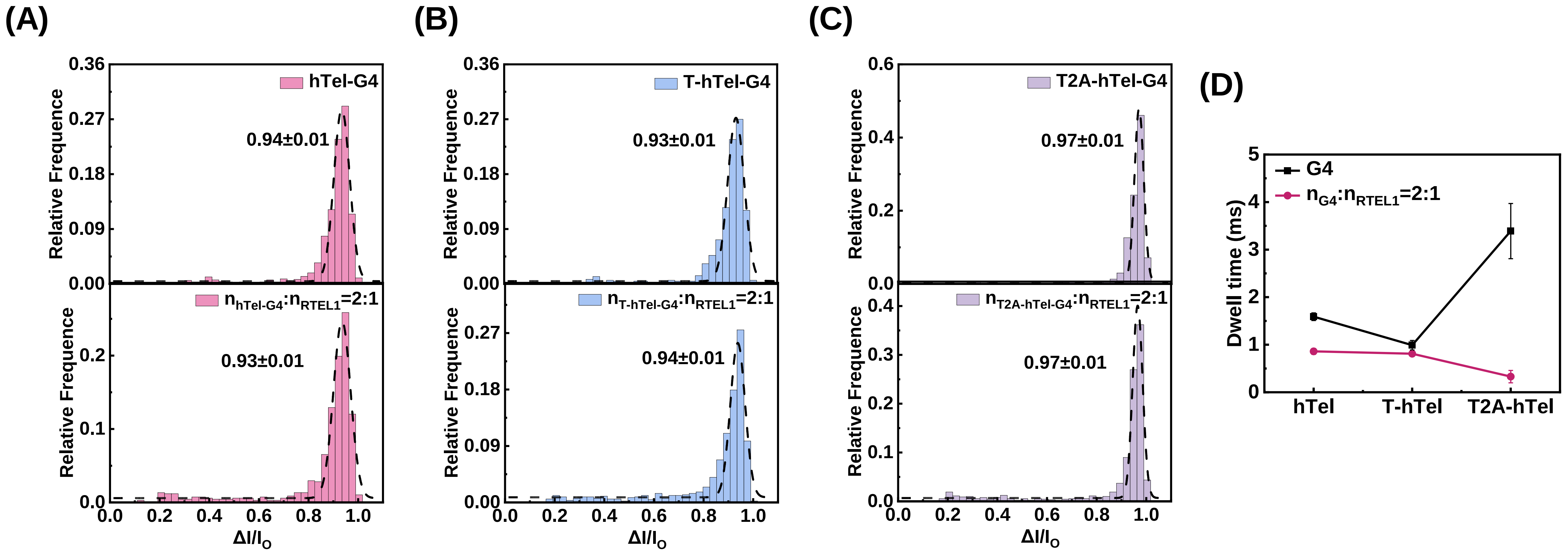

S9. Nanopore translocation properties of G4 and mixed G4 and RTEL1 helicase in 1 M KCl. Histograms of $\Delta I/I_o$ (A-C); line graphs of dwell time (D) of individual G4 and mixed G4 and RTEL1 with molar ratio of 2:1 in 1 M KCl. All the data were recorded with 20 nM hTel/T-hTel/T2A-hTel and mixed hTel/T-hTel/T2A-hTel and RTEL1 with molar ratio of 2:1 for 30 min. in 1 M KCl, Tris, 5 mM ATP, pH 7.4 in a 3.7 nm nanopore under 150 mV.

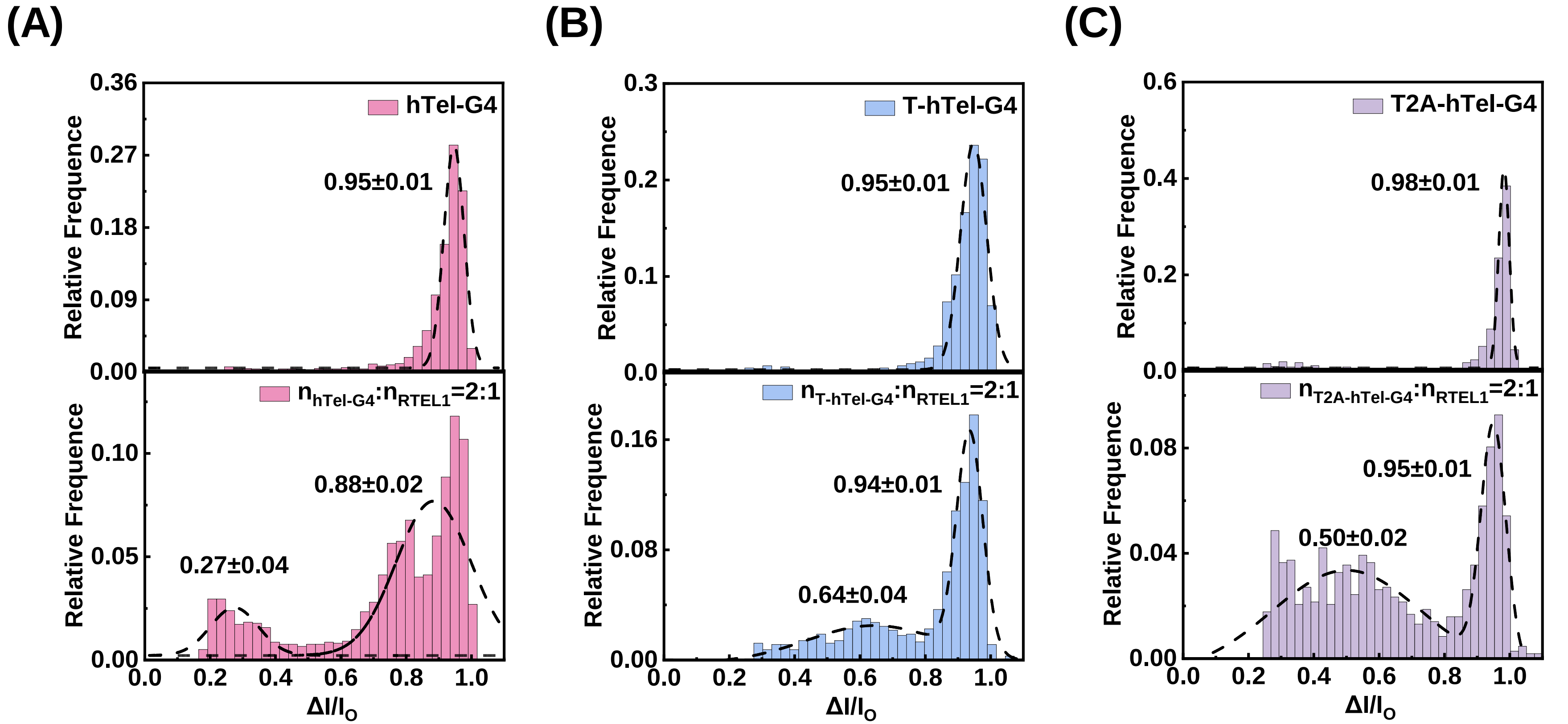

S10. Nanopore translocation properties of G4 and mixed G4 and RTEL1 helicase in 0.5 M CsCl. Histograms of (A-C) $\Delta I/I_o$ of individual G4 and mixed G4 and RTEL1 with molar ratio of 2:1 in 0.5 M CsCl. All the data were recorded with 20 nM hTel/T-hTel/T2A-hTel and mixed hTel/T-hTel/T2A-hTel and RTEL1 with molar ratio of 2:1 for 1 h in 0.5 M CsCl, Tris, 5 mM ATP, pH 5 in a 3.7 nm nanopore under 150 mV.

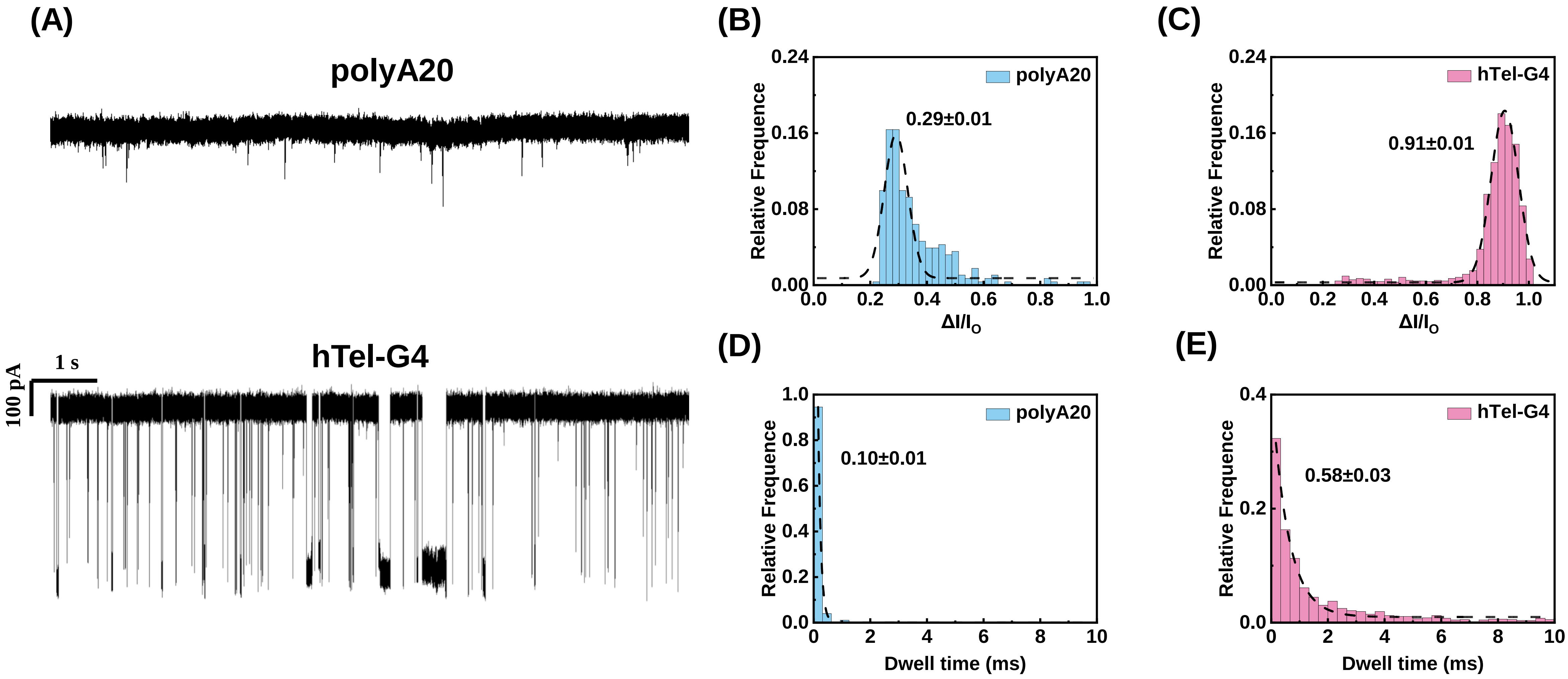

S11. Nanopore detection of homopolymer polyA20 and hTel. (A) Translocation raw traces in 10 s. Histograms of (B-C) $\Delta I/I_o$, (D-E) dwell time. All the data were recorded with 20 nM hTel/polyA20 in 0.5 M CsCl, TE, pH 5 in a 3.7 nm nanopore under 150 mV.

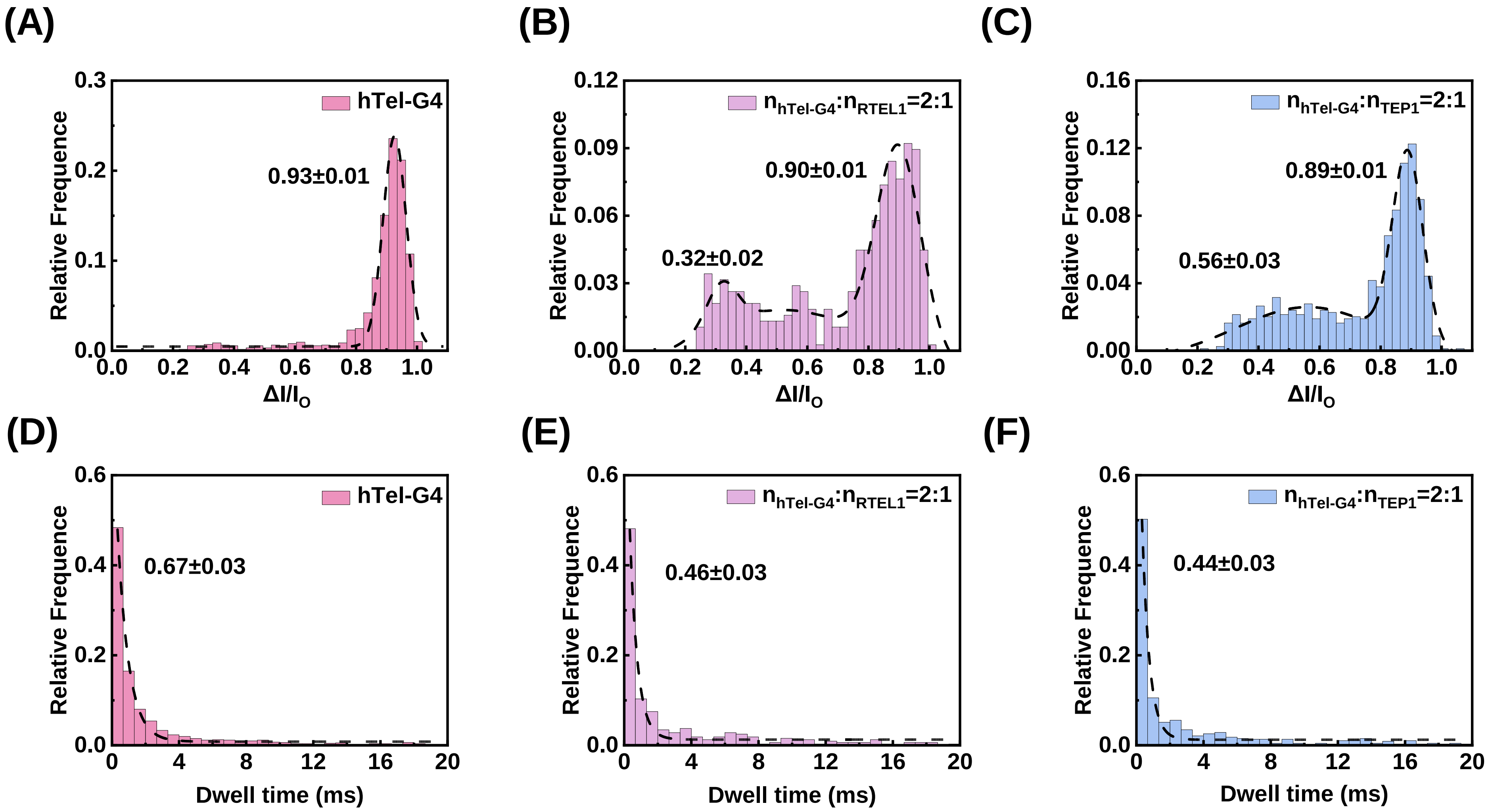

S12. Nanopore-based comparison of hTel-G4 unwinding by RTEL1 and TEP1. Histograms of (A-C) $\Delta I/I_o$, (D-F) Dwell time of individual hTel-G4 and mixed hTel-G4 and RTEL1/TEP1 with molar ratio of 2:1. All the data were recorded with 20 nM hTel and mixed hTel and RTEL1/TEP1 with molar ratio of 2:1 for 1 h in 0.5 M CsCl, Tris, 5 mM ATP, pH 5 in a 3.7 nm nanopore under 150 mV.

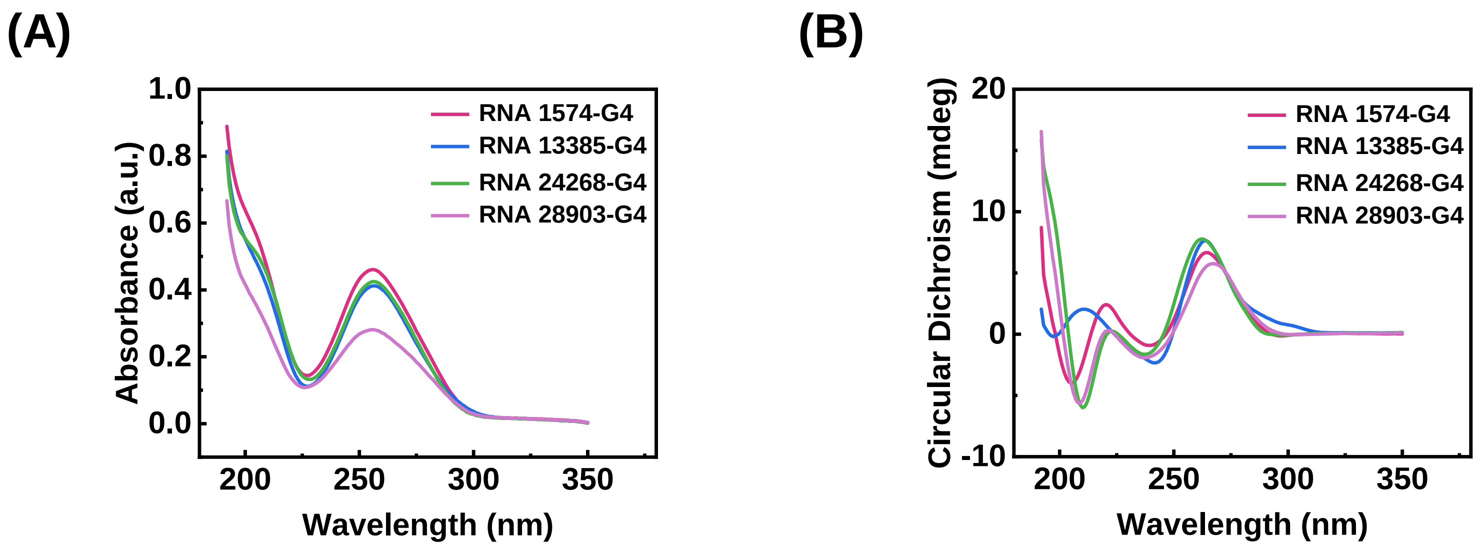

S13. Ultraviolet-visible (UV-Vis) absorption spectra and circular dichroism (CD) spectra of four RNA-G4s. (A) Overlapped UV-Vis absorption spectra; (B) Overlapped Circular dichroism (CD) spectra. Test conditions: 200 μL of 20 μM RNA-G4 in 0.1 M KCl, 10 mM Tris, 1 mM EDTA, pH 7.4.

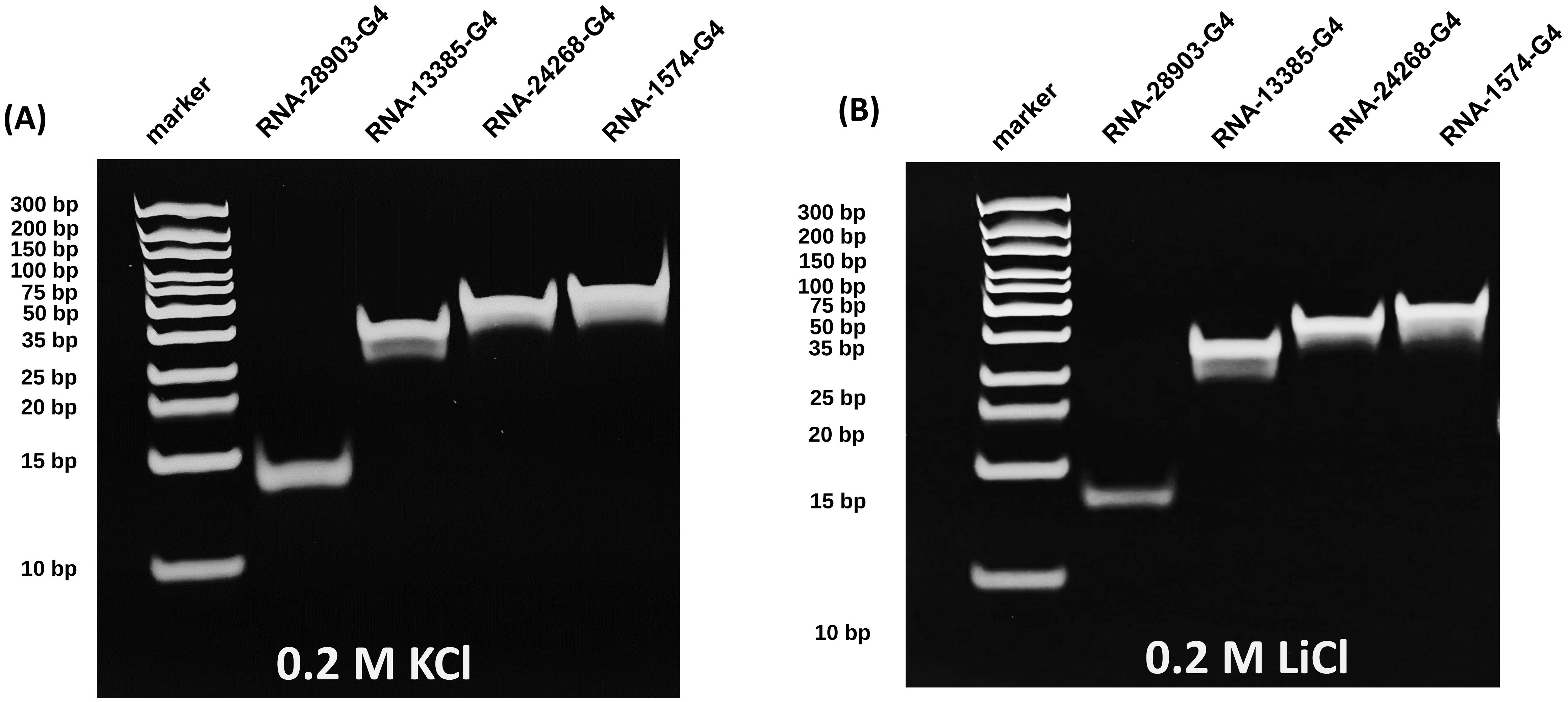

S14. PAGE characterization. 25% of PAGE images of 2 uM RNA-G4 formed in 0.2 M KCl (A) and 0.2 M LiCl (B) with 0.5×TBE as an eluent under 110 V for 2 h.

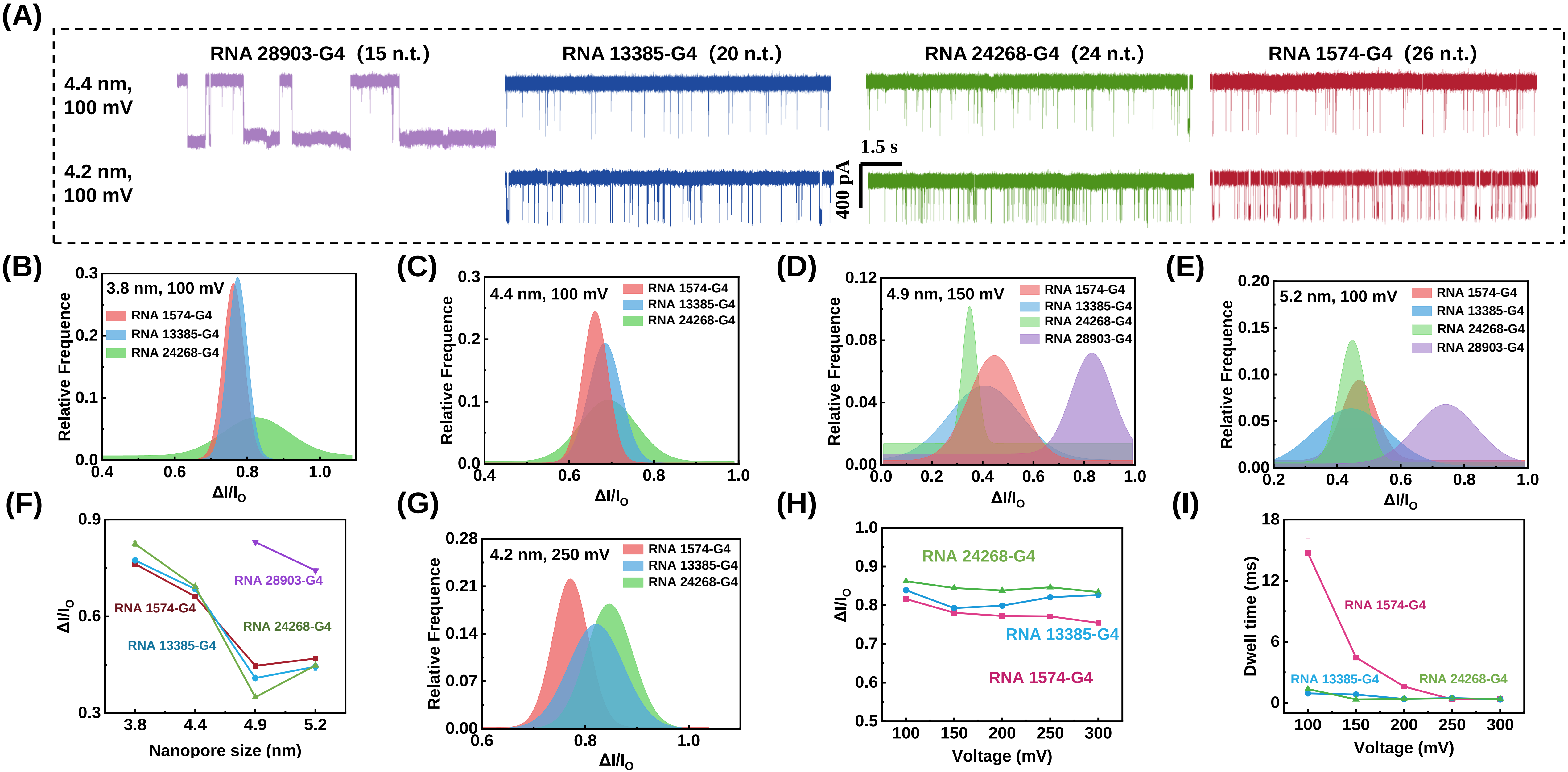

S15. Identification of different RNA-G4 sequences with nanopores. (A) Translocation raw traces in 10 s. Identification of RNA G4 with different nanopore in diameter (B) 3.8 nm, (C) 4.4 nm, (D) 4.9 nm and (E) 5.2 nm (RNA 28903-G4: purple, RNA 13385-G4: blue, RNA 24268-G4: green, RNA 1574-G4: red,). (F) Line graph of $\Delta I/I_o$ as a function of pore diameter. Nanopore discrimination of RNA 1574/13385/24268-G4 (G) Gaussian distribution of $\Delta I/I_o$ at 250 mV, (H) Line graphs of $\Delta I/I_o$ as a function of bias voltages, (I) Line graphs of dwell time as a function of bias voltages. All the data were recorded with 20 nM RNA 1574/ 13385/ 24268/ 28903 in 1 M KCl, TE, pH 7.4. Error bars represent the standard deviation.

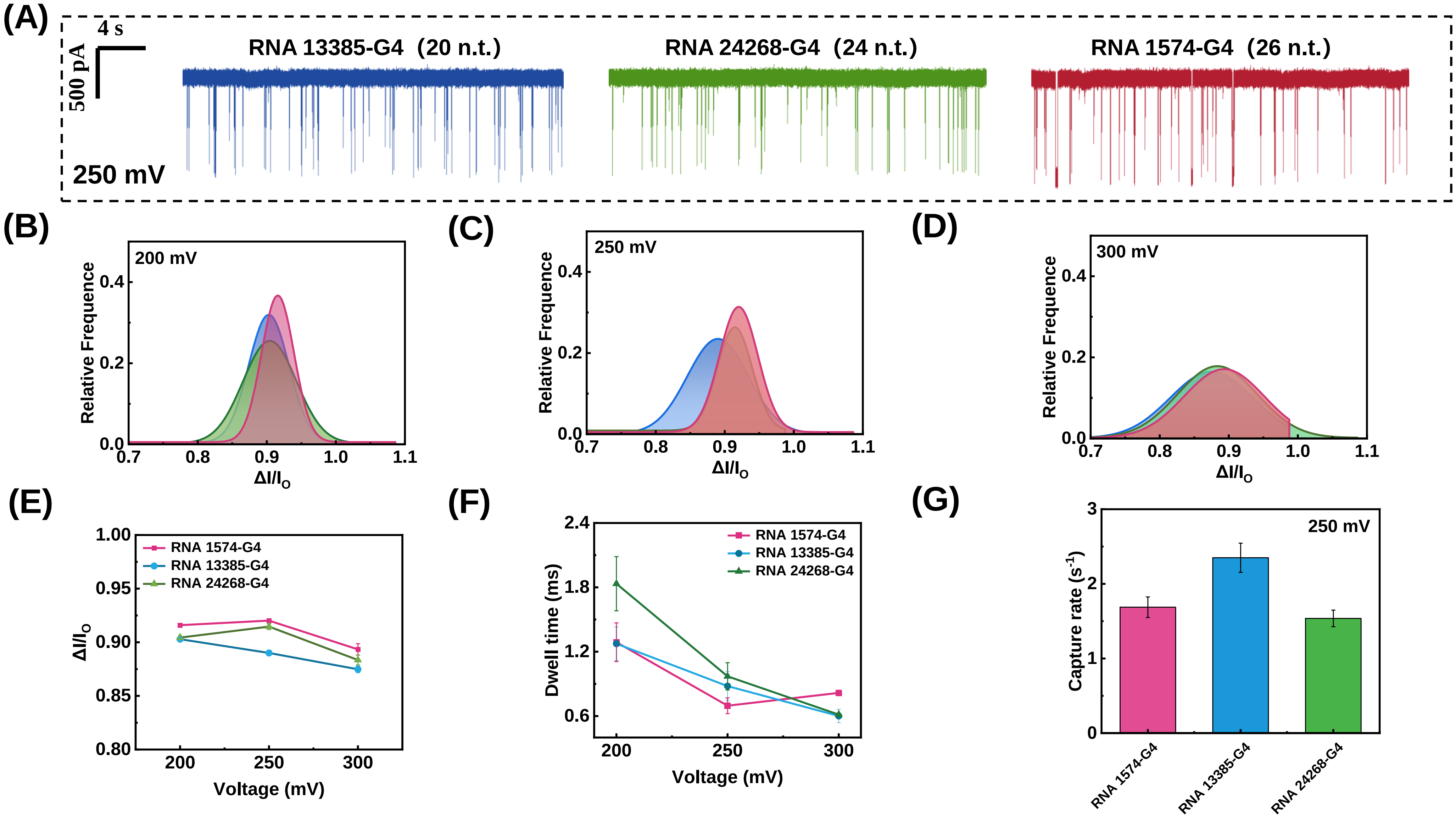

S16. Identification of different RNA G4 sequences with nanopores in 2 M LiCl. (A) Translocation raw traces in 20 s. (B-D) Overlapped gaussian distribution of $\Delta I/I_o$ under distinct bias voltages. (E) Line graphs of $\Delta I/I_o$ as a function of bias potential. (F) Line graphs of dwell time as a function of bias potential. (G) Histograms of capture rate of different G4 sequences at 250 mV. (RNA 1574-G4: red, RNA 13385-G4: blue, RNA 24268-G4: green). All the data were recorded with 10 nM RNA 13385/ 24268/ 1574 in 2 M LiCl, TE, pH 7.4 with a 4.2 nm nanopore. Error bars represent the standard deviation.

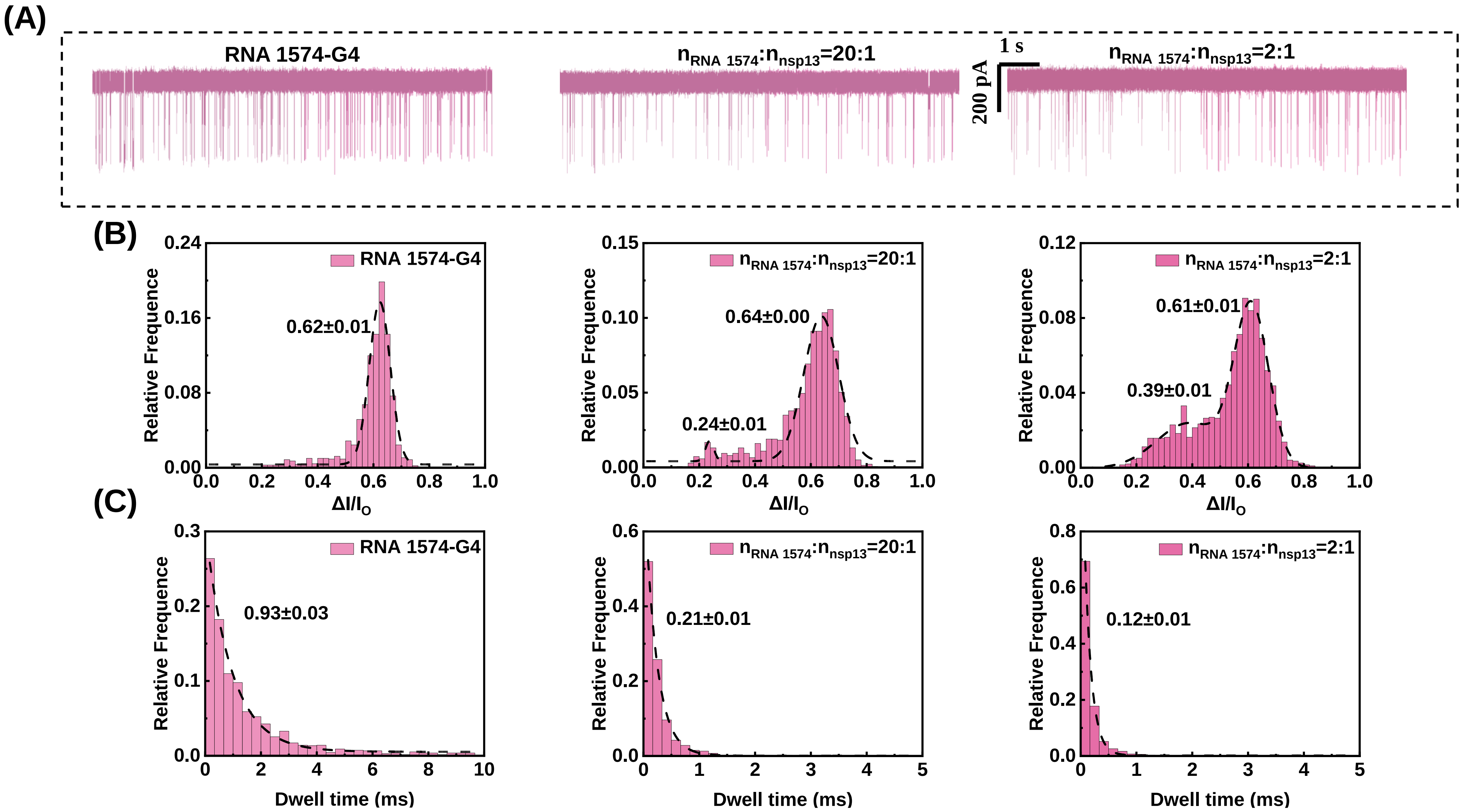

S17. Nanopore translocation properties of G4 and mixed G4 and nsp13 helicase with distinct nsp13 molar ratio. (A) Translocation raw traces in 10 s. Histograms of (B) $\Delta I/I_o$ and (C) blockage duration. All the data were recorded with 10 nM RNA 1574 and mixed RNA 1574 and nsp13 with distinct molar ratio for 30 min. in 0.95 M CsCl/ 0.05 M KCl, TE, pH 7.4 in a 3.6 nm nanopore under 150 mV.

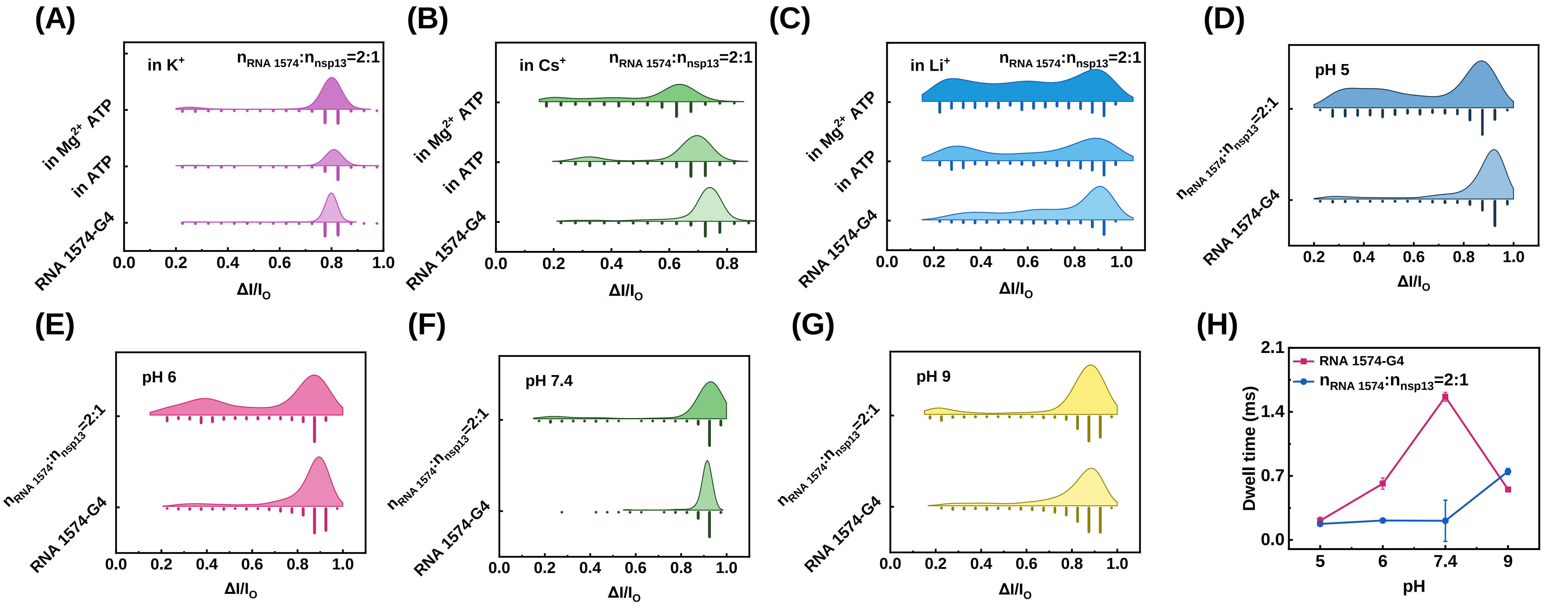

S18. Nanopore translocation properties of G4 and mixed G4 and nsp13 helicase in distinct electrolyte conditions and pH conditions. Assays in different ionic systems of blockage ratio of individual RNA 1574-G4 and mixed RNA 1574-G4, ATP, Mg^{2+} and nsp13 with molar ratio of 2:1 at pH 7.4 in (A) 1 M KCl, (B) 0.5 M CsCl and (C) 2 M LiCl. Assays in different pH conditions of (D-G) blockage ratio and (H) line graphs of dwell time as a function of pH condition for individual RNA 1574-G4 and mixed RNA 1574-G4, along with ATP, Mg^{2+}, and nsp13 at a molar ratio of 2:1. All the data were recorded with 10 nM RNA 1574/ 13385/ 24268 and mixed RNA 1574 and nsp13 with molar ratio 2:1 for 30 min. in 2 M LiCl, Tris, 2 mM $MgCl_2$, 5 mM ATP, in a 3.6 nm nanopore under 100 mV. Error bars represent the standard deviation.

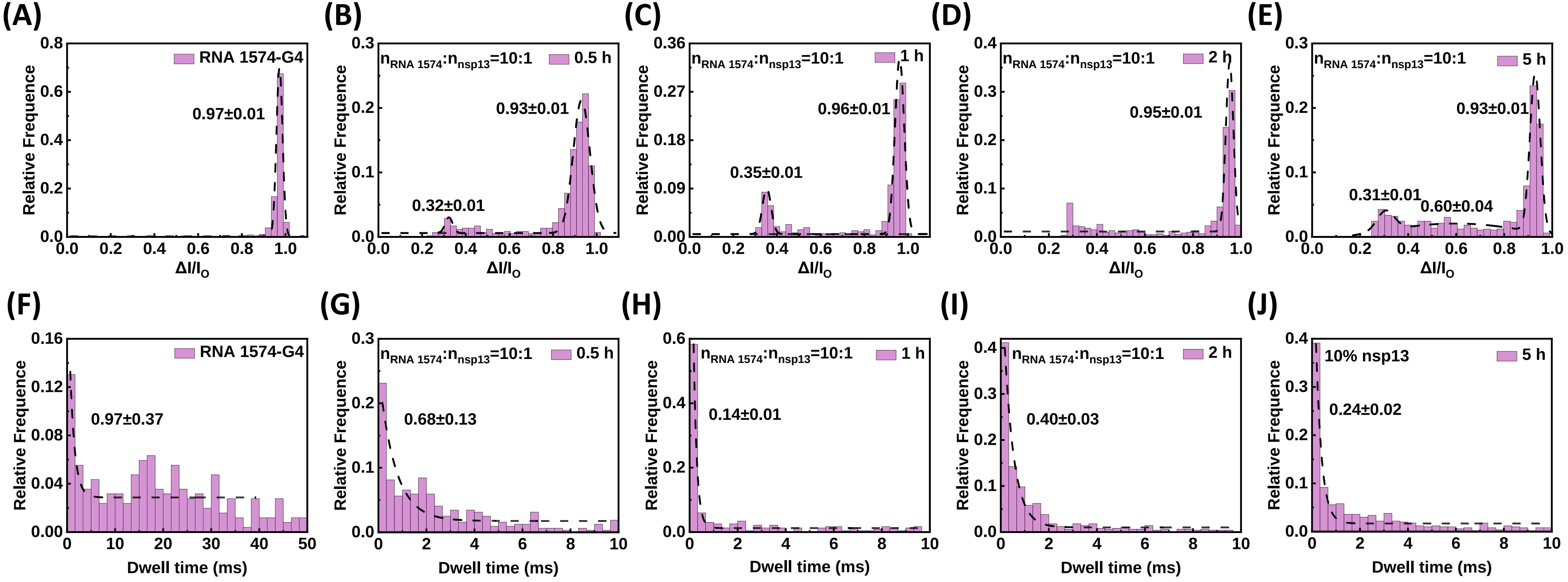

S19. RNA 1574-G4 unwinding process recording with nanopore under distinct incubation time. Histograms of (A-E) blockage ratio, (F-J) blockage duration of individual RNA 1574-G4 and mixed RNA 1574-G4 and nsp13 of molar ratio 10:1 under different incubation time. All the data were recorded with 10 nM RNA 1574 and nsp13 with molar ratio 10:1 in 2 M LiCl, Tris, 2 mM $MgCl_2$, 5 mM ATP, pH 5, in a 3.6 nm nanopore under 100 mV.

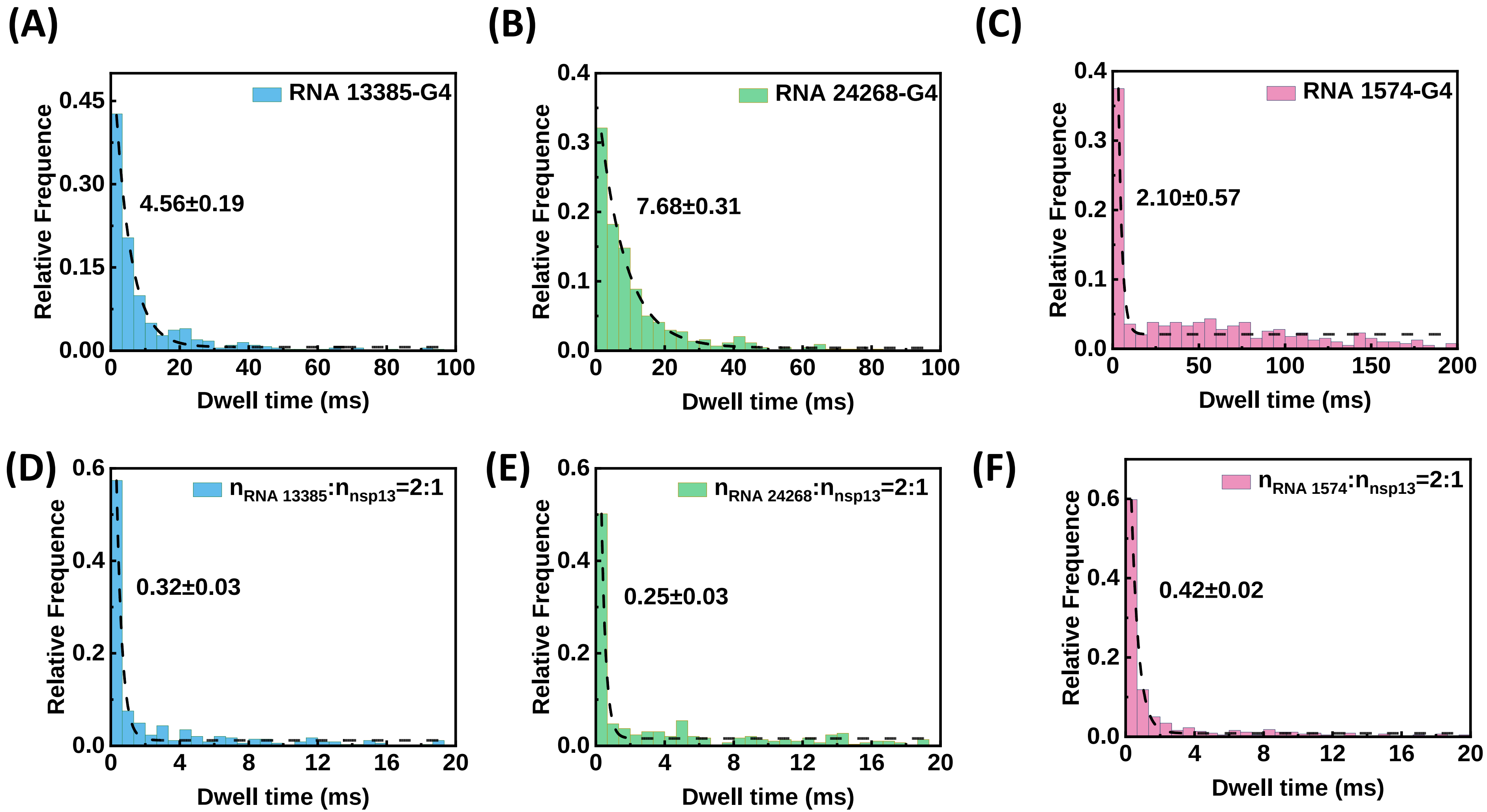

S20. Unfolding selectivity of nsp13 with G4 of distinct topologies. Histograms of blockage duration of the translocation of (A-C) individual G4 and (D-F) mixed G4 and nsp13 with molar ratio 2:1. All the data were recorded with 10 nM RNA 1574 and nsp13 with molar ratio 2:1 for 1 h in 2 M LiCl, Tris, 2 mM $MgCl_2$, 5 mM ATP, pH 5 in a 3.6 nm nanopore under 100 mV.
